# Effect of Ropivacain and Bupivacain on Calcium‐Related and G‐Protein Coupled Processes in PMNs: A Human In‐Vitro Study

**DOI:** 10.1002/hsr2.71636

**Published:** 2025-12-09

**Authors:** Richard Felix Kraus, Thies Galla, Michael Gruber, Sigrid Wittmann

**Affiliations:** ^1^ Department of Anesthesiology University Hospital Regensburg Regensburg Germany

**Keywords:** G‐protein‐coupled receptors, local anesthetics chemotaxis, neutrophil function, signal transduction

## Abstract

**Background and Aims:**

We investigated the impact of altered intracellular calcium levels and G‐protein‐coupled receptor (GPCRs) signaling inhibition on migration and NETosis of polymorphonuclear leukocytes (PMNs) under influence of bupivacaine and ropivacaine.

**Methods:**

PMNs were isolated from whole blood of healthy volunteers by centrifugation. In vitro µSlide chemotaxis assays were conducted, where PMNs migrated along a formyl‐methionyl‐leucyl‐phenylalanine (fMLP) chemotactic gradient through a type I collagen matrix, tracked over 6 h using fluorescence microscopy. Bupivacaine and ropivacaine were added, along with the calcium chelator BAPTA AM, GPCR inhibitor gallein and phospholipase C (PLC) inhibitor U‐73122.

**Results:**

In contrast to ropivacain, bupivacaine induced earlier NETosis. Both local anesthetics caused an earlier cessation of PMN migration. Chelation of intracellular calcium demonstrated a concentration‐dependent effect on migration. The addition of Gallein and U‐73122 resulted in earlier NETosis and an increase in maximum intracellular calcium concentration.

**Conclusion:**

Intracellular calcium appears to play a minimal role in the process of NETosis, while it is significantly important for neutrophil migration. Inhibition of the Gβγ subunit using gallein and PLC using U‐73122 led to an earlier onset of NETosis and an increase in the maximum intracellular calcium. An additional effect of ropivacaine on the GPCR signaling pathway was not detectable.

## 1 Introduction

Local anesthetics frequently used in everyday clinical practice (like bupi‐ and ropivacaine) are able to influence the functional properties of PMNs via diverse mechanisms [1, 2]. G protein‐coupled receptors (GPCRs) and intracellular calcium levels are considered as important interfaces that affect neutrophil functions such as migration and NETosis [1, 3].

**Figure 1:**
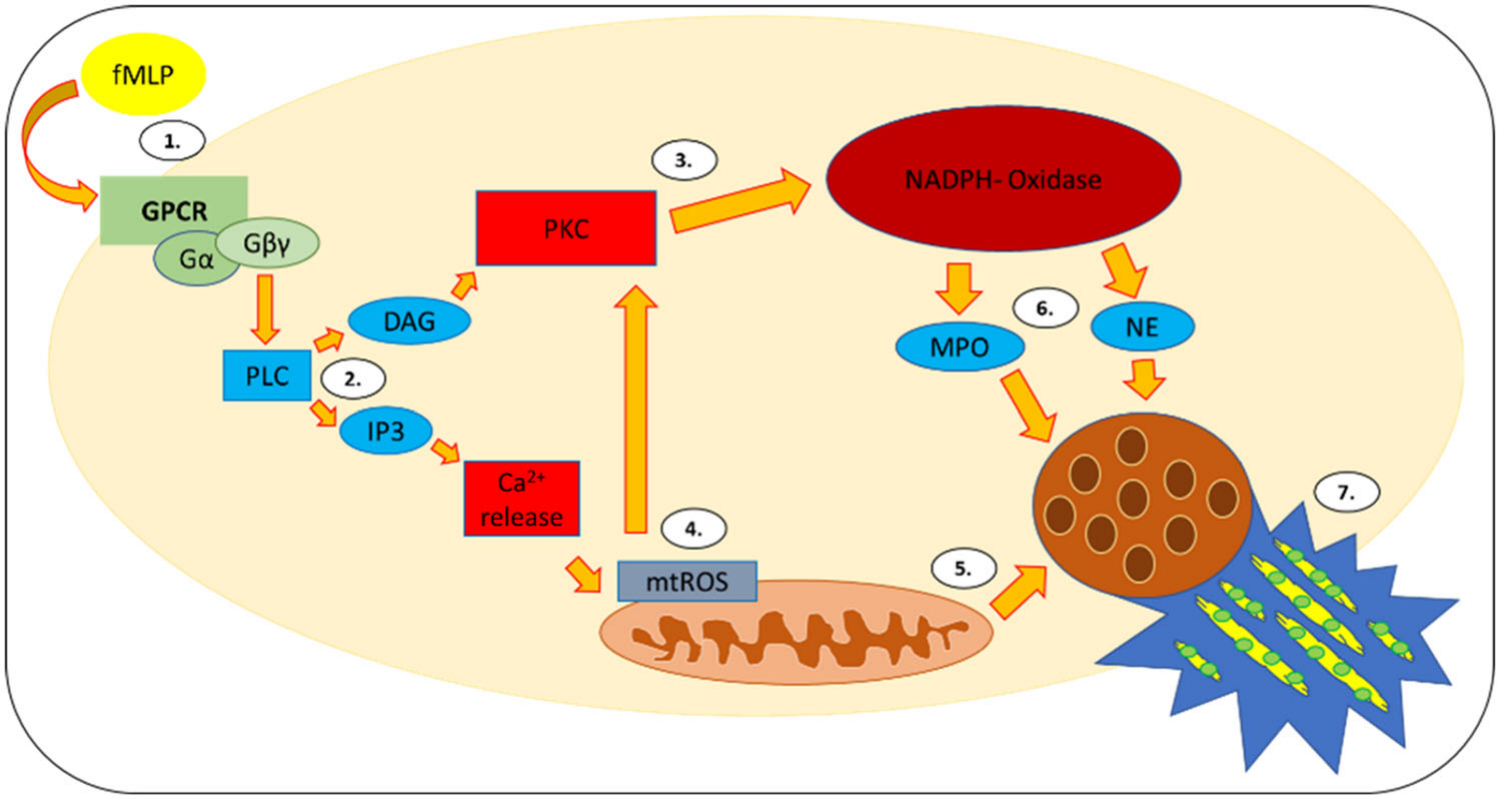
NETosis cascade: The activation of the G protein‐coupled receptors (GPCR) via formyl‐l‐methionyl‐leucyl‐phenylalanine (fMLP) (1) leads to the triggering of the intracellular signaling cascade of phospholipase C (PLC) (2) with release of diacylglycerol (DAG) and inositol‐1,4,5‐trisphosphate (IP3). IP3 increases the intracellular calcium concentration, which leads to the initiation of mitochondrial production of reactive oxygen species (ROS) (4). DAG triggers protein kinase C (3), which stimulates nicotinamide‐adenine‐dinucleotide‐phosphate (NADPH) ‐oxidase. Subsequently, myeloperxosidase (MPO) and neutrophil elastase (NE) (6) are released, which, just as mitochondrial ROS (mtROS) (5), can cause NETosis (7) by reaching the cell nucleus. (Figure is an own illustration provided by Co‐Author Thies Galla [8]).

Migration and NETosis can be triggered by stimuli such as lipopolysaccharides (LPS), N‐formyl‐l‐methionyl‐leucyl‐phenylalanine (fMLP) or chemokines [4, 5, 6, 7]. NETosis is triggered intracellularly by protein kinase C (PKC), and activates NADPH oxidase (NOX) at the end of the signaling cascade (Figure 1) [7, 9]. Moreover, the Raf/MEK/ERK but also the p38 or PI3K/AKT signaling pathway, which are activated by protein kinases, are also involved in NETosis [4, 6, 10, 11].

In addition, there is a NADPH oxidase‐independent NETosis, which is associated with increased intracellular calcium concentrations and the resulting influence on potassium channels (SK3). However, these channels only increase mitochondrial production of reactive oxygen species (ROS) but not that of NOX [4, 12]. There is evidence that fMLP can activate NOX in the granules via direct and indirect activation of the specific enzyme segment NOX2, and that mitochondrial ROS production is part of the mechanism [7, 13, 14].

Increased neutrophil ROS production is triggered by activation of the GPCR and is associated with an increased release of intracellularly stored calcium. This occurs in a NOX‐dependent and NOX‐independent way through stimulation with fMLP [15]. Generally, the increase in ROS production leads to reduced stability of the granular and nuclear membranes.

In the past, it was demonstrated that NETosis but also migration can be influenced by local anesthetics under septic conditions [1, 16]. A change in intracellular calcium concentration [17] as well as molecular inhibition or stimulation of PMN surface proteins (like upregulation of activated complement C3 receptor; C3aR) are discussed as triggering mechanisms [18, 19].

PMNs can be activated via a GPCR by the tripeptide fMLP [20, 21]. The kinases PI3K and PLC, which are triggered via the Gβγ subunit of the GPCRs, are known to be part of the signaling cascade inducing NETosis [18, 22, 23]. An interaction between intracellular calcium and these kinases might exist in PMNs [6, 7].

Calcium is an essential second messenger that transduces a multitude of intracellular responses of PMNs stimulated via GPCR and tyrosine kinase receptors [17]. Calcium is involved in oxidative response and cytokine secretion [24, 25] and is part of the NETosis signaling cascade. The effect of intracellular calcium depends on its duration, amplitude, frequency and also on spatial localization [17].

**Figure 2:**
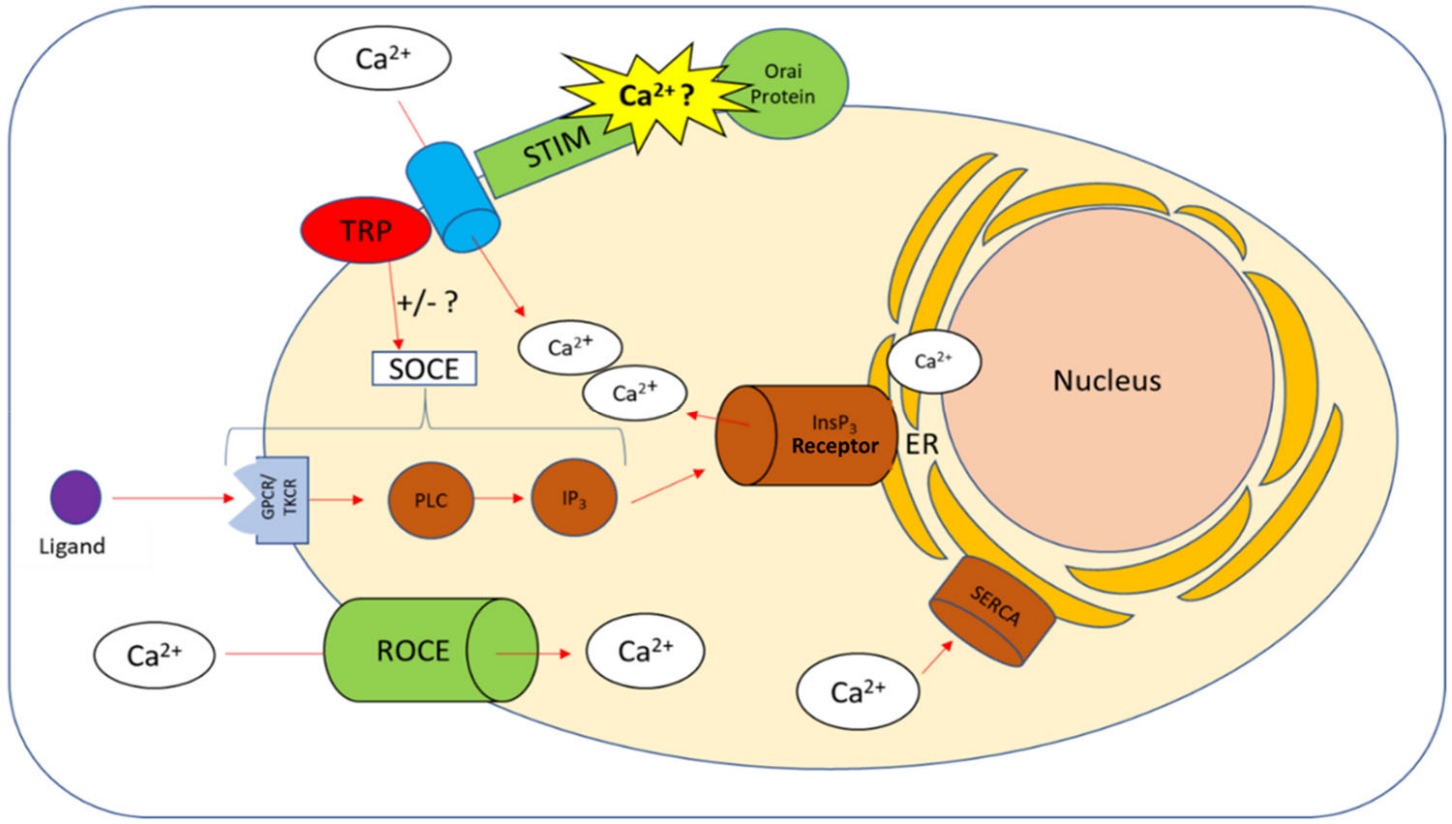
Transport mechanisms of calcium in the PMN. The intracellular calcium balance of the PMN is maintained by the intracellular calcium store or by influx from the extracellular milieu [17]. Regulation takes place via transporters and signaling pathways such as store‐operated calcium entry (SOCE), receptor‐operated calcium entry (ROCE) and sarcoplasmic reticulum (SERCA) [24]. SERCA pumps intracellular calcium into the endoplasmatic reticulum (ER), which is the most important calcium store [26]. SOCE is a signaling pathway activated via a G protein‐coupled (GPCR) and a tyrosine kinase‐coupled receptor (TKCR) [17]. Via these receptors, ligands activate a phospholipase C/inositol 1,4,5‐triphosphate (IP3) signaling pathway [27]. IP3 binds to the specific receptor InsP3 on the ER causing it to release its calcium store. This process stimulates calcium influx through the SOCE located in the plasma membrane [17]. SOCE is mediated by the stromal interacting molecules (STIM), the Orai protein, a calcium channel and the transient receptor potentials (TRP). This is a ternary complex in which the STIMS have the function of a calcium sensor [24, 28, 29]. ROCEs are receptors that serve the influx of extracellular calcium independently of the intracellular calcium depot [17, 30]. (Figure is an own illustration provided by Co‐Author Thies Galla [8]).

The intracellular calcium store of PMNs is mainly located in the endoplasmic reticulum (ER, Figure 2). In addition, there is a small store in the mitochondria. The storage level of the ER is regulated via the Ca^2+^ ATPase of the sarcoplasmic reticulum (SERCA), as well as by inositol 1,4,5‐triphosphate receptors, ryanodine receptors (RYRs) and calcium‐binding proteins such as calreticulin and calsequestrin [27, 31, 32]. RYRs have only recently become known as components of PMNs. Earlier studies could only prove their existence in skeletal muscle (RYR type 1), cardiac muscle (RYR type 2) and the brain (RYR type 3) [33].

Local anesthetics exert an effect on voltage‐dependent sodium channels, but also surface structures of the PMN such as GPCRs [3]. These structures are specifically targeted for the treatment of autoimmune diseases, ARDS, sepsis or tumor growth and metastasis. The common feature of these pathologies is the formation of NETs by PMNs [34, 35]. No voltage‐dependent sodium channels could be detected in PMNs [35]. Thus, the effects triggered by local anesthetics, such as NETosis, must be controlled by other mechanisms [36, 37]. The research team led by Futosi et al. described numerous surface structures of PMNs. These include GPCRs [18]. In 2004, Hollmann et al. described in their in vitro and in vivo experiments that local anesthetics are able to inhibit GPCRs under certain exposure times and different concentrations. In vivo, effects were seen even at low, clinically relevant concentrations (1–10 µM). In vitro, a similar effect could only be achieved with a 100‐fold higher concentration of the local anesthetic [38]. They also demonstrated that an inhibition of lysophosphatidic acid (LPA) by ropivacaine is possible [39]. LPA is a GPCR anchored in the cell membrane, which stimulates different intracellular signaling cascades such as, for example, cell proliferation [40].

**Figure 3:**
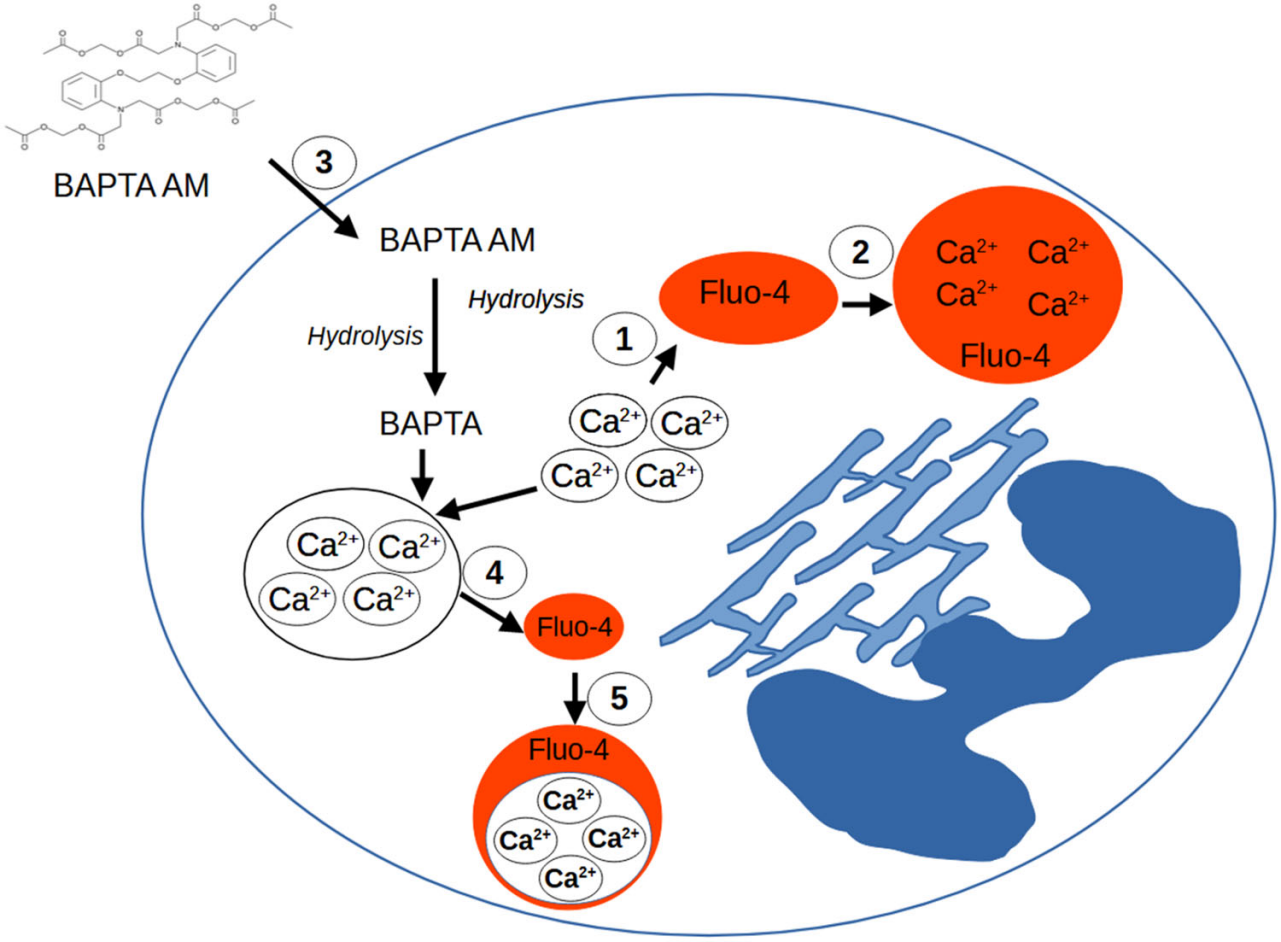
Reaction process with BAPTA AM: ① Up to 4 cytosolic calcium‐ions are bound by the fluorescent dye Fluo‐4. ② As a result, the Fluo‐4‐Ca^2+^ complex fluorescence intensity rises (microscopic visible). ③ BAPTA AM permeates intact cell membranes and is cleaved by cytosolic esterases to BAPTA ④ When BAPTA is present in the cytosol, the concentration of free Ca^2+^ ‐ ions is reduced. ⑤ The concentration of Fluo‐4‐Ca^2+^ complexes is therefore reduced as well. The fluorescence intensity decreases. *BAPTA AM and Fluo‐4 were experimentally (artificially) added from the outside and are not naturally present in the cell and are also not released by cells. (Figure is an own illustration provided by corresponding author Richard Kraus, modified according to [8]).

The objective of our study was to clarify whether local anesthetics have effects on intracellular calcium or other intracellular signaling cascades as well as receptors of PMNs and thus influence their migration and NETosis. For this purpose, PMNs were isolated from the whole blood of volunteers. Chemotaxis experiments (live cell imaging) were conducted in which the effects of bupivacaine and ropivacaine as well as of the calcium chelator BAPTA AM were tested. BAPTA AM ester permeates the intact cell membrane and is intracellularly cleaved by esterases to BAPTA which is trapped in the cell and chelates cytosolic calcium (Figure 3).

**Figure 4:**
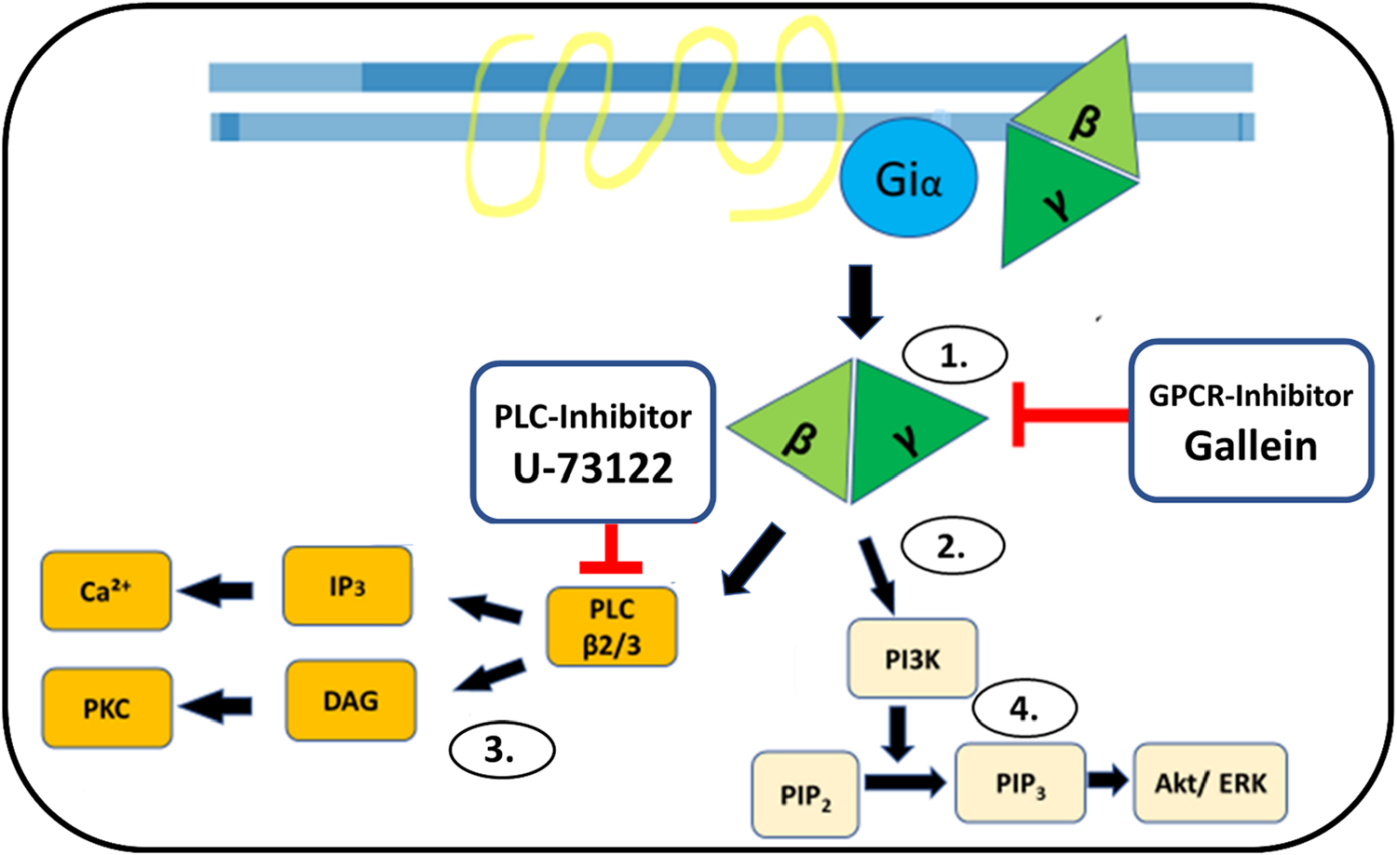
Effect of GPCR inhibitors: ① Activation of a GPCR is interrupted at the Gβγ subunit by the GPCR inhibitor Gallein or Phospholipase C (PLC) inhibitor U‐73122). ② As a result, the further signaling cascade via PLC or PI3K does not take place. Blocking of: ③ PLCß2/3 stimulation of IP3 and DAG. Blocking of: ④ PI3K activation of the Akt/ERK signaling pathway. (Figure is an own illustration provided by Co‐author Thies Galla, modified by Richard Kraus according to [8]).

In addition, it was examined whether GPCR antagonist binding has an effect on PMNs. For this purpose, the Gβγ subunit of the GPCR was inhibited during the activating transition to PI3K and the PLC signaling pathway. The GPCR inhibitor gallein was used to inhibit PI3K while the PLC signaling pathway was inhibited by U‐73122 (Figure 4).

## 2 Methods

### 2.1 Sample Collection

The conduct of this study was approved by the local Ethics Committee of the Medical Faculty of the University of Regensburg (file number 12‐101‐0192). After informed consent venous puncture was performed in volunteers´ (*n* = 51) antecubital fossa or on the back of the hand to obtain 10 mL of blood using a Safety‐Multifly® needle with sizes 0.8×19 mm or 0.9×38 mm (Sarstedt AG & Co., Nümbrecht, Germany). The blood sample was collected in a lithium heparin monovette (Sarstedt AG & Co.).

### 2.2 PMN Isolation by Density Gradient Centrifugation

**Figure 5:**
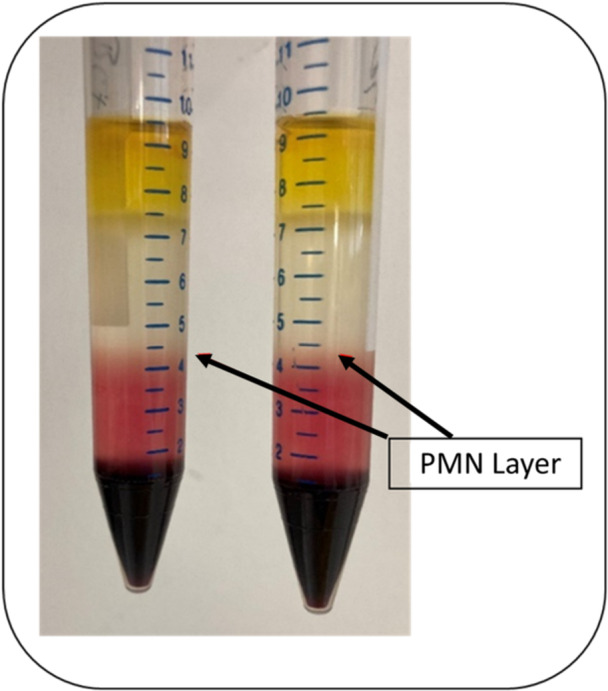
Different cell layers after density gradient centrifugation. The arrows mark the layer containing the PMNs.

PMNs were isolated by density gradient centrifugation [41]. 3 mL of Leuko Spin Medium (Pluri Select Life Science, Leipzig, Germany) was layered with 3 mL of Lympho Spin Medium (Pluri Select Life Science) in two 15 mL centrifuge tubes. This was followed by careful layering of 3 mL whole blood of each sample. The cell separation was performed by 20 min centrifugation (with Thermo Scientific™ Biofuge™ Stratos™ centrifuge (Heraeus Sepatech, Hanau, Germany)) at room temperature at a speed of 756 G without centrifuge brake. Centrifugation of the sample material was used to separate the blood into several phases with the aim of obtaining a consistent PMN ring (Figure 5).

The layers above the PMN ring were aspirated and discarded. Subsequently, 300 µL of each PMN ring was collected and placed in a 15 mL centrifugation tube. The PMN suspension was diluted with 3 mL PBS (Dulbecco′s Phosphate Buffered Saline modified without calcium, magnesium and chloride, ThermoFisher Scientific, Massachusetts, USA). 10 µL of this dilution was taken to determine the number of PMNs using a Neubauer counting chamber. Then, the cell suspension was centrifuged at 272 *g* for 5 min at room temperature. The supernatant was aspirated and resuspended with the calculated volume of cell culture medium RPMI 1640 (PAN‐Biontech GmbH, Aidenbach, Germany) containing 10% fetal calf serum (FKS, Sigma‐Aldrich Chemie GmbH, Steinheim, Germany) to reach a final concentration of 18 × 10^6^ cells/mL.

### 2.3 In Vitro Chemotaxis Model: Live Cell Imaging

**Figure 6:**
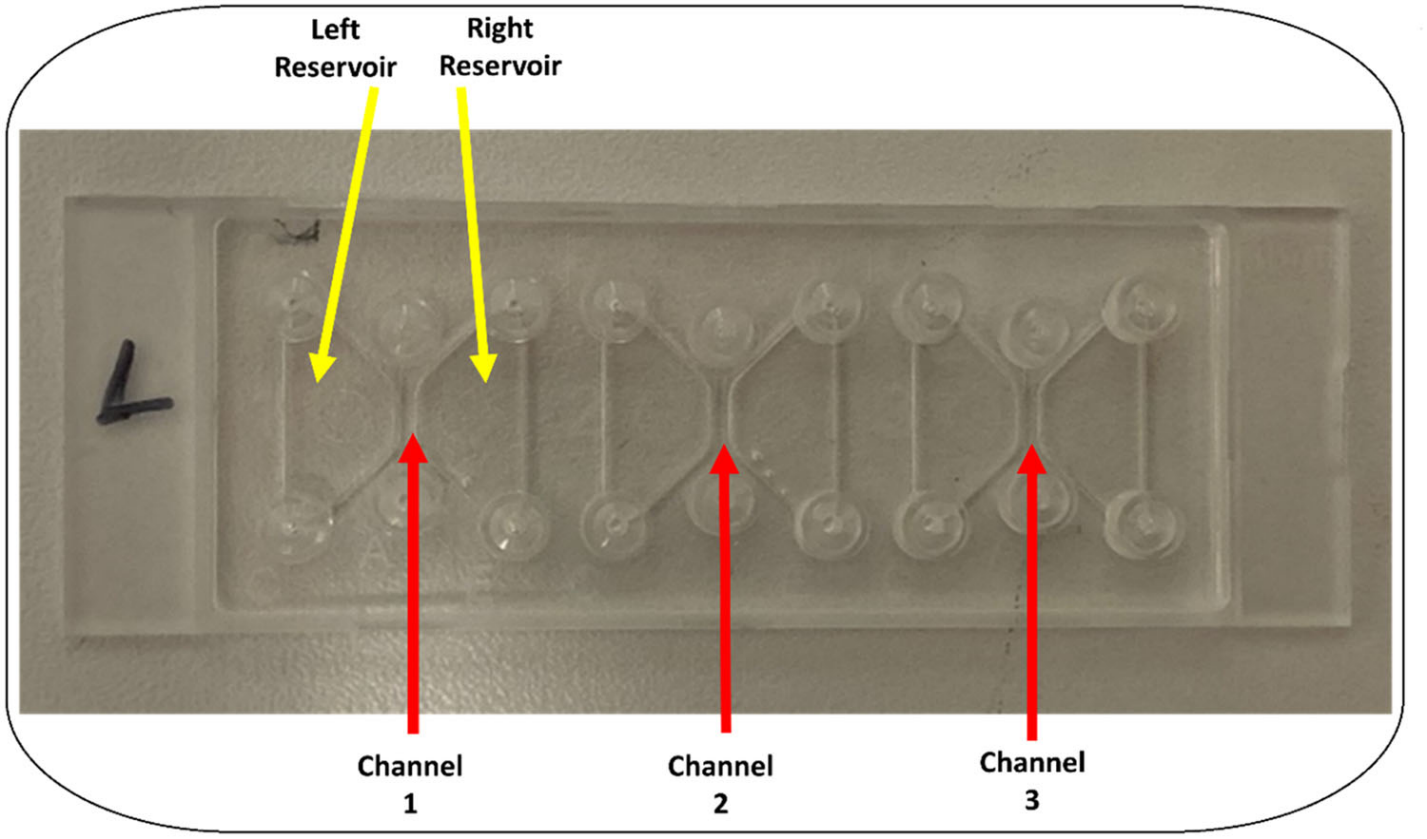
Structure of the IBIDI® 3D µ‐slide chemotaxis chambers.

For experimental observation of neutrophil chemotaxis and functional PMN analysis, 3D‐µ‐slide chemotaxis chambers from IBIDI® (IBIDI® GmbH, Planegg/Martinsried, Germany) were used. The structure of each 3D‐µ‐Slide consists of three chambers. These consist of a channel and two reservoirs each (Figure 6).

The channels were filled with a 1.5 mg/mL bovine collagen‐I matrix, which consisted of the components listed in Table 1.

**Table 1:** Components of the matrix production medium.

Component	Volume [µL]
10× MEM (Minimum Essential Medium, M0275, Sigma Aldrich)	20
Distilled water	20
NaHCO_3_^−^ (Emsure® ACS, Merck KGaA, Darmstadt, Germany)	10
RPMI 1640 (PAN‐ Biotech GmbH, Aidenbach, Germany	50
PureCol® (Advanced BioMatrix Inc. San Diego)	150
Cell suspension + fluorescent dyes	50

A total of *n* = 51 experiments (i.e. *n* = 51 blood samples and *n* = 51 µSlide chambers with 3 channels each) were performed. Each of the 3 channels was filled with a 1.5 mg/mL type I collagen matrix as a basis. In each channel, 4′, 6‐diamidino‐2‐phenylindole dihydrochloride (DAPI, D9542‐5MG, Sigma‐Aldrich Chemie GmbH, Steinheim, Germany) was used to visualize NETosis at a concentration of 5 µM [41, 42]. Fluo 4‐Fluorescent labeling reagent (Abcam, Berlin, Germany) was used at a concentration of 30 µM to monitor the intracellular calcium (Figure 3) and thus the calcium concentration in connection with the NETosis and migration taking place [43].

On top of that, different additions were made to this matrix (the exact filling procedure is described in the sections below).

**Figure 7:**
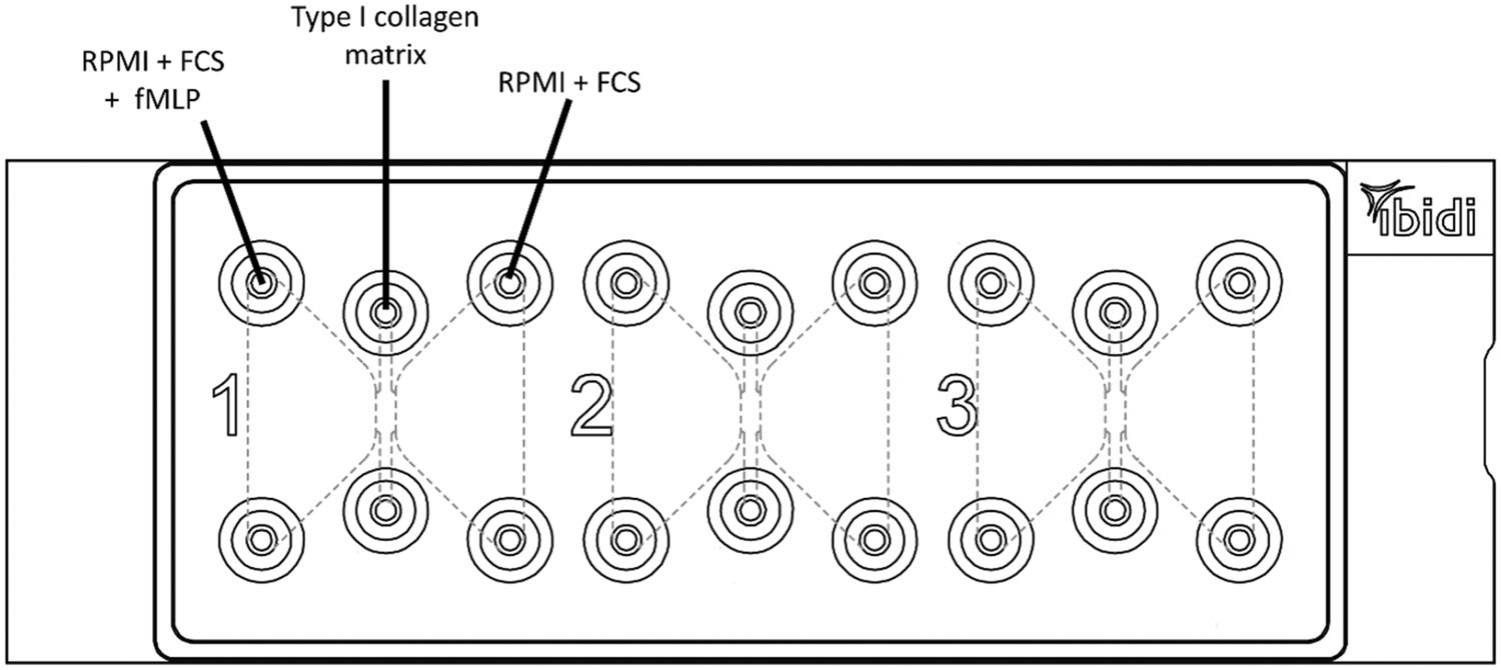
Basic filling of the µSlide chambers: All channels were filled with type I collagen in all channels, RPMI + fetal calf serum (FCS) in all reservoirs and fMLP in all left reservoirs (here schematically labeled on the left).

In each experiment, all reservoirs of all 3 positions were filled with the basic medium RPMI 1640 + 10% fetal calf serum, whereby in each left reservoir an fMLP solution (with final 10 nM concentration) was pipetted (Figure 7). In some experiments local anesthetics were added to the reservoirs (see below). The exact number of the distinct experiments, which were valid for analysis, is listed in the tables of the supplement.

### 2.4 Ca2+ Chelatation and Antagonisation of GPCR and PLC

#### 2.4.1 First Test Series

**Figure 8:**
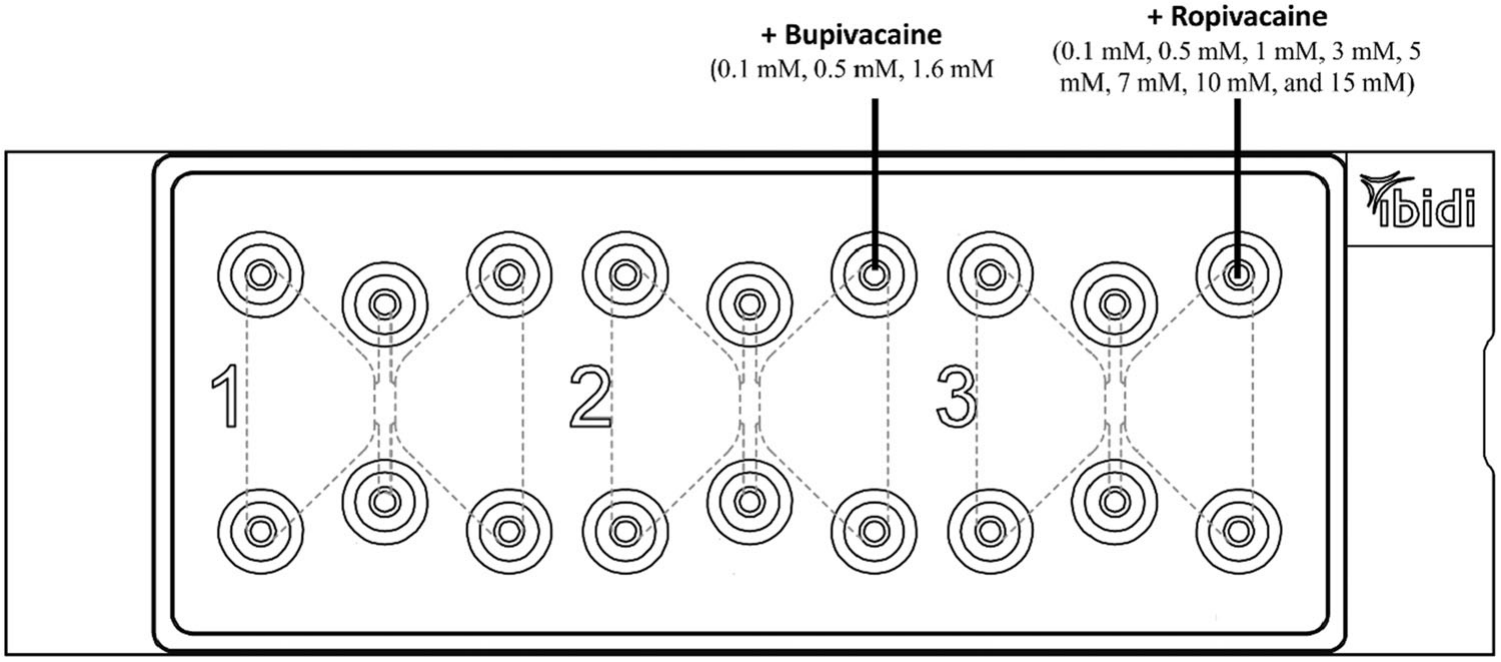
Filling of the first test series: local anesthetics were filled additionally in the reservoirs of channel 2 + 3.

In the first test series, no addition was made to the type I collagen matrix, but the reservoirs of the middle and the right channel were additionally filled with bupivacain (channel 2) and ropivacaine (channel 3). Ropivacaine was used in concentrations of 0.1 mM, 0.5 mM, 1 mM, 3 mM, 5 mM, 7 mM, 10 mM, and 15 mM; bupivacaine was used at concentrations of 0.1 mM, 0.5 mM, and 1.6 mM (Figure 8).

#### 2.4.2 Second Test Series

**Figure 9:**
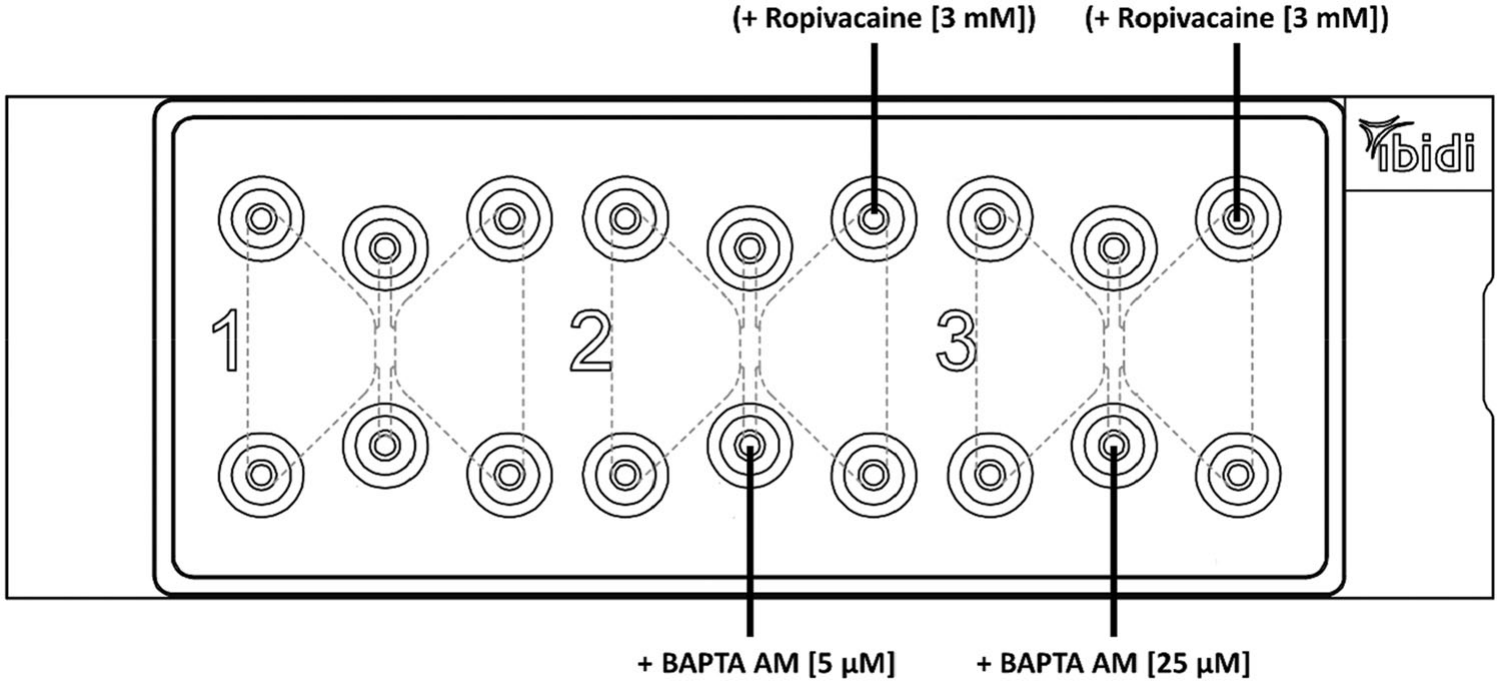
Filling of the second test series: BAPTA AM was filled additionally in channel 2 + 3 and in some experiments ropivacaine in the right reservoirs.

In the second test series, BAPTA AM was added to matrix (channel 2 + 3) [17]. Channel 1 was used as control without BAPTA AM. BAPTA AM final matrix concentration was 5 µM in channel 2 and 20 µM in channel 3 [44]. In some experiments of 2nd series ropivacaine [3 mM] was added to the right reservoir (Figure 9).

#### 2.4.3 Third Test Series

**Figure 10:**
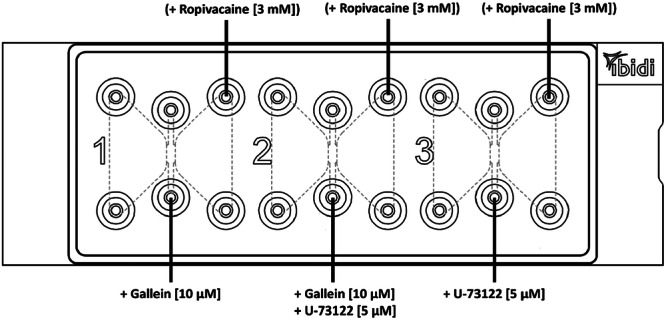
Filling of the third test series: Gallein was added in channel 1 and U‐73122 was added in channel 3. In channel 2 Gallein and U‐73122 both were added and in some experiments ropivacaine in the right reservoirs.

In the third test series, Gallein (pyrogallol phthalein) was added in channel 1 and U‐73122 was added in channel 3. In channel 2 Gallein and U‐73122 both were added. Gallein was used at a concentration of 10 µM and U‐73122 at 5 µM [45, 46, 47]. For this purpose, a 10,003 µM Gallein stock solution and a U‐73122 stock solution with a concentration of 5244 µM were prepared. Both were dissolved in DMSO. The desired final concentration of DMSO was 0.1% in all three channels in order to create similar conditions in all inhibitor concentrations. In some experiments of 2nd series ropivacaine [3 mM] was added to the right reservoir (Figure 10).

### 2.5 Microscope Setup for Life Cell Imaging

PMN function was observed over a period of 14 h using a Leica DMi8 microscope (Leica Microscopy & System GmbH, Wetzlar, Germany) with 100x magnification. Leica Application Suite X software (LAS X 3.0.4.16529, Leica Mikroskopie & System GmbH, Wetzlar, Germany) was used for microscope control. The Leica DFC9000 GT camera (Leica Microscopy & System GmbH, Wetzlar, Germany) and the CoolLED pE4000 exposure system (CoolLED Ltd., Andover, England) were used for analysis and electronic documentation. One image sequence was taken every 30 s. The exposure energy was 90%. The selected filter and dye properties are shown in the following Table 2.

**Table 2:** Details of fluorescent‐microscopic observation.

	DAPI	Anti‐MPO‐APC	Fluo‐4
Wavelength of maximum excitation [nm]	359	650	510
LED wavelength used [nm]	385	635	490
Inlet filter [nm]	380–410	615−635	472−498
Wavelength of maximum emission [nm]	461	660	532
Outlet filters used [nm]	420–460	650	505−545

### 2.6 Data Analysis

The image data sets were processed using Imaris® software (Bitplane AG, Zurich, Switzerland). The analyzed information was tabulated using Excel and then further evaluated:

For migration evaluation, individual cells were recognized and the following migration parameters were recorded (Table 3). The total of 14 h of observation was divided into 30‐min time slots.

**Table 3:** Parameters recorded for quantifying migration via live‐cell‐imaging.

Parameter	Definition
Track Displacement X = TDX [µm]	Distance on the x‐axis
Track Displacement Y = TDY [µm]	Distance on the y‐axis
Track Displacement Length = TDL [µm]	Distance between the first and last position of the cell (=euclidean distance)
Track Length = TL [µm]	Total covered migration length
Track Speed Max [µm/s]	Maximum speed for covering the distance
Track Speed Mean [µm/s]	Average speed to cover the distance
Track Speed Min [µm/s]	Minimum speed for covering the distance
Track Duration [s]	Duration between the first and last time within a track
Track Straightness [D/L]	Measure for target of migration

The different fluorescent dyes (see Section 2.3) foregrounded different cellular events (NETosis, MPO release and intracellular calcium changes), which was recognized by Imaris. For NETosis, NETosis confirmation with MPO and the intracellular calcium concentration, the “Surfaces” were recorded using Imaris® software and the data sets for: Area, Birth [s], Death [s], Time points [s] and fluorescence area sums. The data sets were analyzed using Phoenix® software (Certara L.P., Radnor, PA, USA).

### 2.7 Statistics

Statistical analysis was performed using the statistical program SPSS® Version 26 (SPSS® IBM® Company, Armonk, NY, USA). All groups were subjected to the Kolmogorov‐Smirnov test for normal distribution. In the case of multiple comparisons, the significant difference between the groups was tested with the one‐factorial analysis of variance (ANOVA) in case of normal distribution. Variance homogeneity was assessed according to Bonferroni and variance heterogeneity according to Dunnett T3. The statement about the variance was verified using the Levene test. If the normal distribution was not given in the individual groups, the central tendency of the individual groups to be tested was tested according to Kruskal‐Wallis. This was then also followed by the analysis of variance. The Mann‐Whitney U‐test was used to examine the central tendency of two independent samples for which no t‐test could be used due to the lack of a normal distribution. The results were presented graphically using simple or grouped box plots. The outliers were represented as dots or asterisks outside the upper and lower whiskers. Statistical significance was defined as a probability of error of *p* < 0.05 [48].

## 3 Results

### 3.1 Characteristics of the Test Persons

A total of 51 participants (34 females, 17 males) were included in this study. The median age of the participants was 23 years, the median height 172 cm, the median weight 63 kg.

### 3.2 Standardization of the Fluorescent Measurements

**Figure 11:**
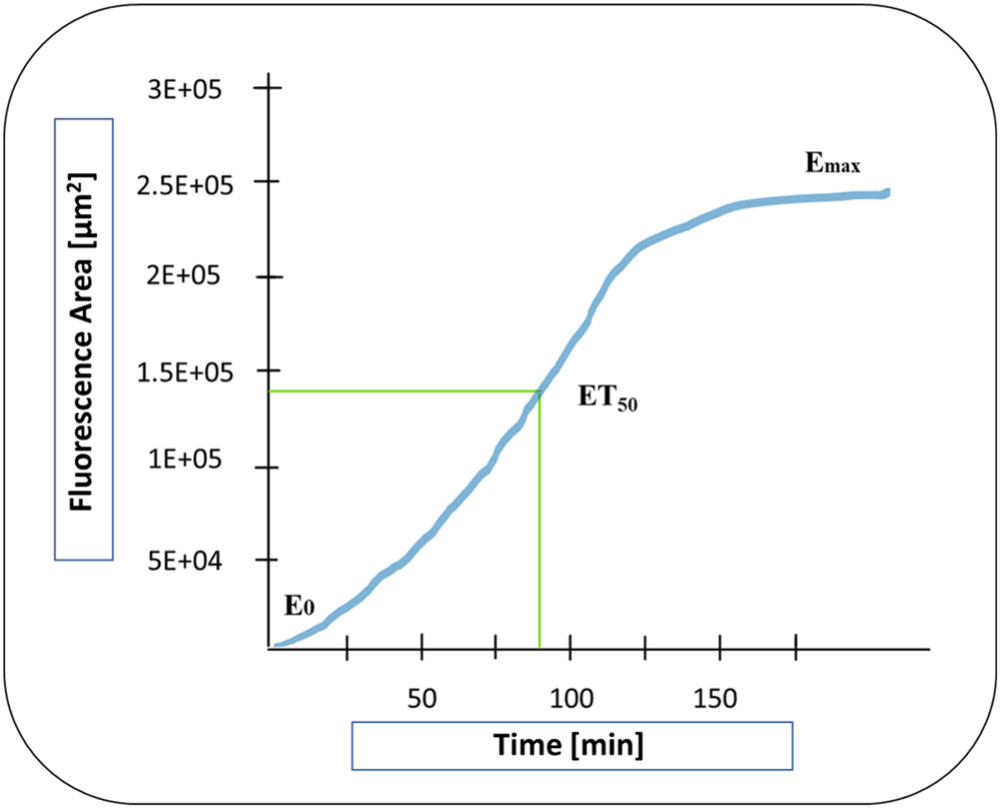
Measurement of the time point when the half‐maximum effect is reached ET_50_ (ET_50_NETosis, ET_50_MPO).

The analysis of NETosis and MPO fluorescence revealed sigmoidal curves. Therefore, the half‐maximum growth of the maximum area sum (ET_50_NET [min], ET_50_MPO [min] und IT_50_Fluo‐4 [min]) was determined (see Figure 11).

**Figure 12:**
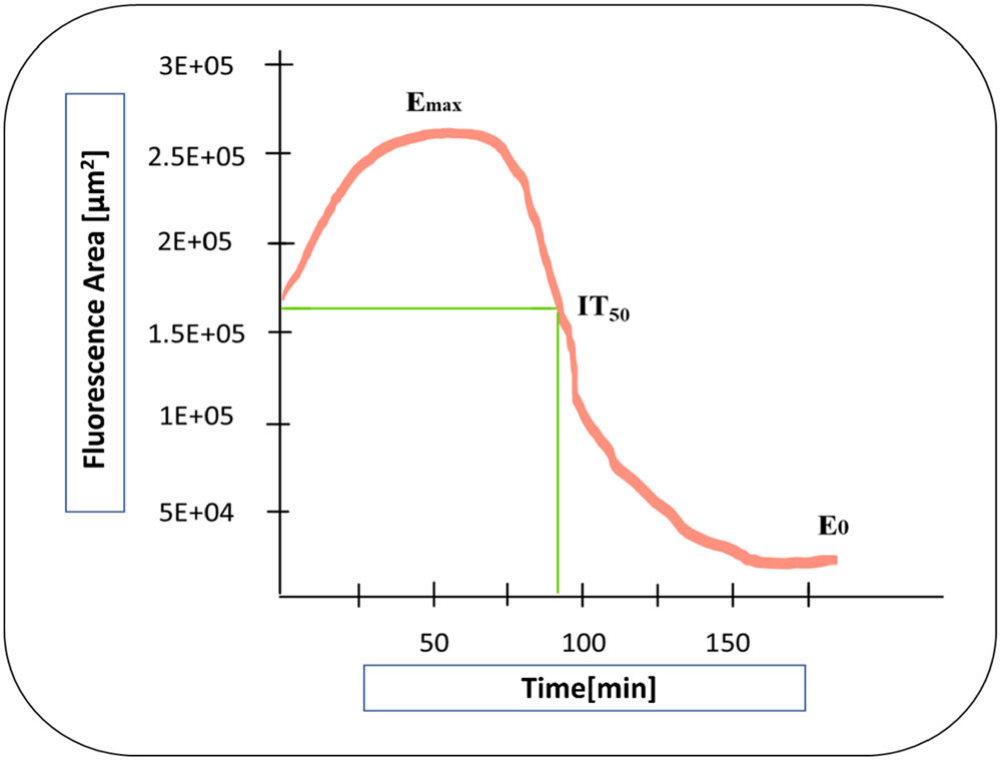
Measurement of the time point when the maximum effect has declined to 50% IT_50_ (IT_50_Fluo‐4).

The course of IT_50_Fluo‐4 [min] showed an opposite sigmoidal course than ET_50_NETosis [min] and ET_50_MPO [min]. In addition, this inhibitory sigmoidal curve showed a time point of maximum Fluo‐4 activity and thus the maximum intracellular calcium concentration at a given time point (see Figure 12).

### 3.3 Influence of Local Anesthetics on NETosis and Intracellular Calcium Levels

#### 3.3.1 Effect of Bupivacaine and Ropivacain on ET_50_NETosis

**Figure 13:**
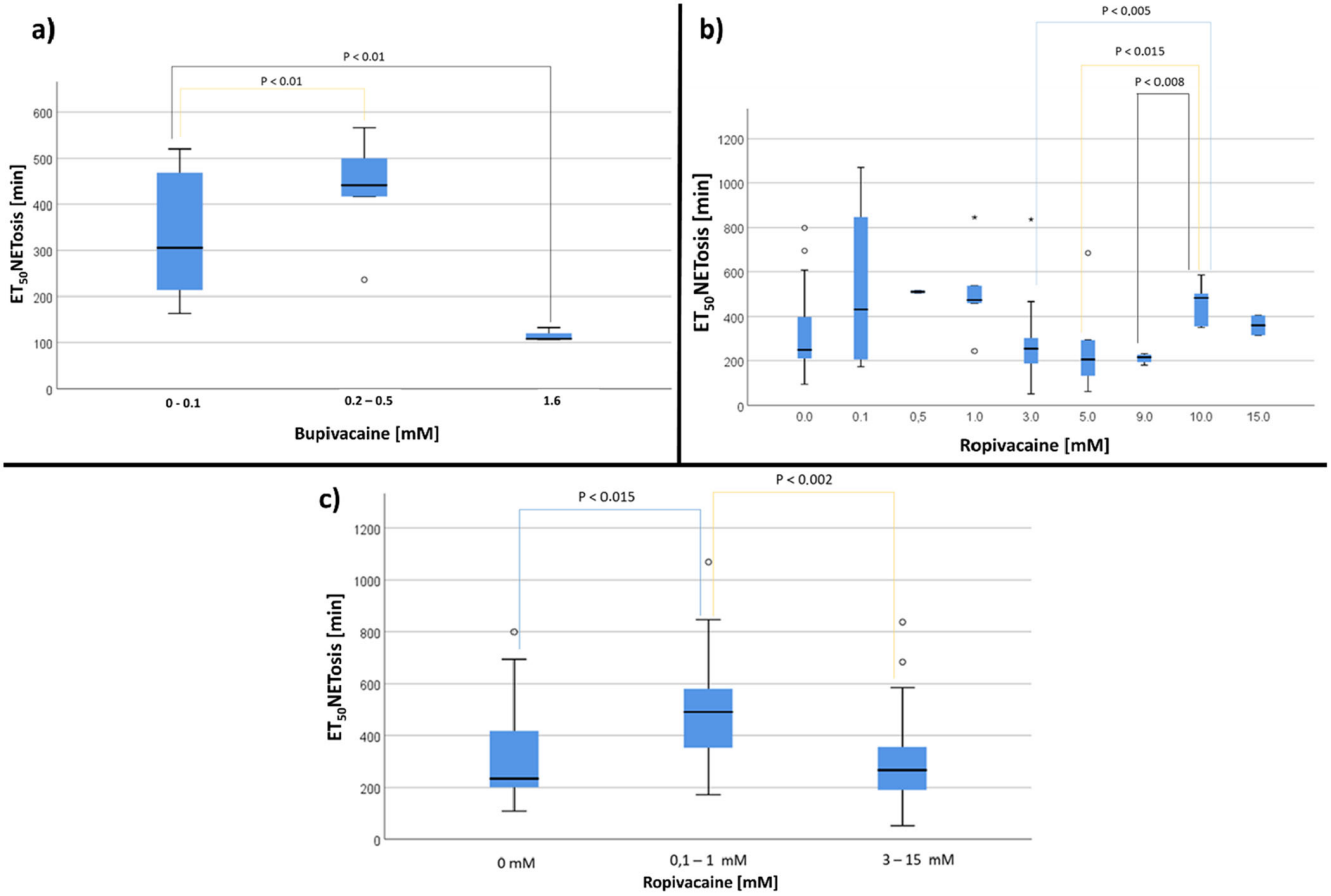
(a) Influence of bupivacaine on ET_50_NET [min]. (b) Influence of different Ropivacaine concentrations on ET_50_NET [min]. Authors' note: Parts of these results have already been published [49]. (c) Comparison of grouped ropivacaine concentrations ((0 mM, 0.1–1 mM and 3–15 mM) on ET_50_NETosis [min]. Data is shown as median with interquartilrange.

The comparison of ET_50_NET [min] of the 0.1 mM concentration group with the 0.5 mM and 1.6 mM bupivacaine concentration group revealed significant differences (Figure 13a and Table S1). The medium concentrations of ropivacaine (3 mM, 5 mM and 9 mM) showed partly earlier NETosis (ET_50_NETosis) compared to the low concentrations (0.1 mM, 0.5 mM and 1 mM), yet without reaching significance. At high concentrations of ropivacaine (10 mM, 15 mM), NETosis was delayed compared to NETosis at medium concentrations (3 mM, 5 mM, 9 mM). Significant differences were also detected between NETosis at medium concentrations 3 mM, 5 mM and 9 mM compared to NETosis at high concentration (10 mM, Figure 13b and Table S2). ET_50_NETosis values of 15 mM ropivacaine group did not differ significantly from the values of the 3 mM, 5 mM and 9 mM concentration group. Additionally, ET_50_NETosis at concentrations 0.1–1 mM and ≥ 3 mM of ropivacain were summarized and compared without the addition of ropivacaine. Significant differences could be observed (Figure 13c and Table S3).

#### 3.3.2 Influence of Intracellular Calcium Chelation and GPRC Inhibition on NETosis

**Figure 14:**
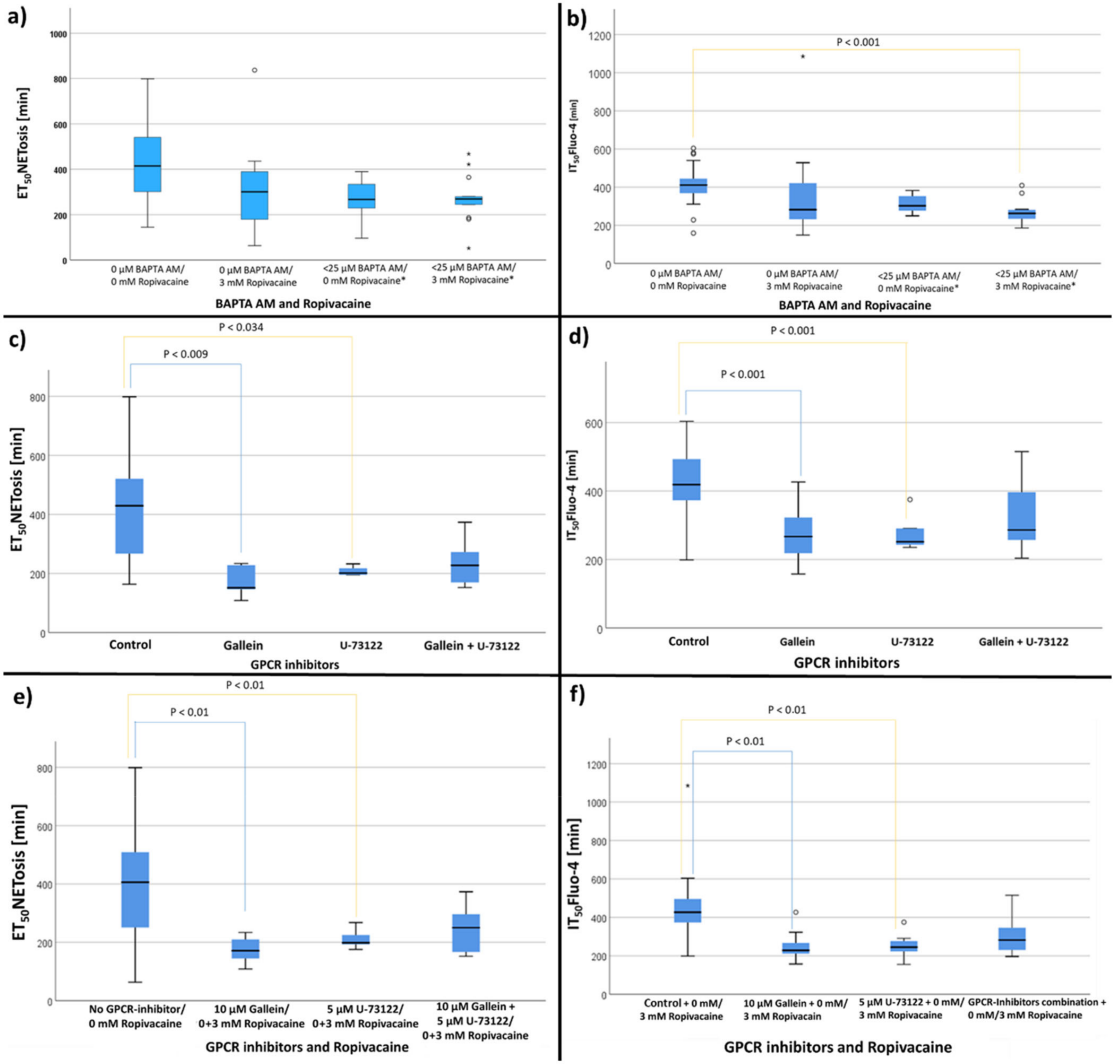
(a) Influence of BAPTA AM and ropivacaine on ET_50_NETosis [min]. Data is shown as median with interquartile range. (b) Influence of BAPTA AM and ropivacaine on IT_50_Fluo‐4 [min]. (c) Influence of GPCR inhibitors on ET_50_NETosis [min]. (d) Influence of GPCR inhibitors on IT_50_Fluo‐4 [min]. (e) Influence of GPCR inhibitors and ropivacaine on the ET_50_NETosis [min]. (f) Comparison of GPCR inhibitors and ropivacaine concentrations. Data is shown as median with interquartile range. * < **25 µM** = **5–25 µM BAPTA‐AM**.

No significant effect of BAPTA AM and ropivacaine on ET_50_NETosis [min] was observed (Figure 14a and Table S4). IT_50_Fluo‐4 of combined BAPTA AM (5−25 µM) and ropivacaine (3 mM) was significant earlier than of the control (Figure 14b and Table S5). The experiments conducted with 30 µM BAPTA‐AM did not generate any analyzable results. ET_50_NETosis of Gallein and U‐73122 was significant earlier than of the control. No difference of ET_50_NETosis was observed between the control group and the combined addition of gallein and U‐73122 (Figure 14c and Table S6). IT_50_Fluo‐4 values of GPCR inhibitors Gallein and U‐73122 were significant earlier than of the control (Figure 14 d and Table S7).

There was no significant difference between ET_50_NETosis of the control, gallein, U‐73122 and gallein + U‐73122, when 3 mM ropivacaine was present. Therefore, the groups of the control with and without 3 mM ropivacaine were combined with each other and the groups of gallein, U‐73122 and their combination. Herein, ET_50_NETosis of Gallein and U‐73122 were significant earlier than of the control group (Figure 14e and Table S8). No differences were observed for the comparison of IT_50_Fluo‐4 of the control group with the IT_50_Fluo‐4 results of Gallein, U‐73122 each. Control groups with and without 3 mM ropivacaine were summarized. The same approach was used for the groups of Gallein, U‐73122, and their combinations. IT_50_Fluo‐4 was significant earlier for Ropivacaine in combination with Gallein as well as U‐73122 (Figure 14f and Table S9).

### 3.4 PMN Migration

#### 3.4.1 Influence of Bupivacaine and Ropivacaine

**Figure 15:**
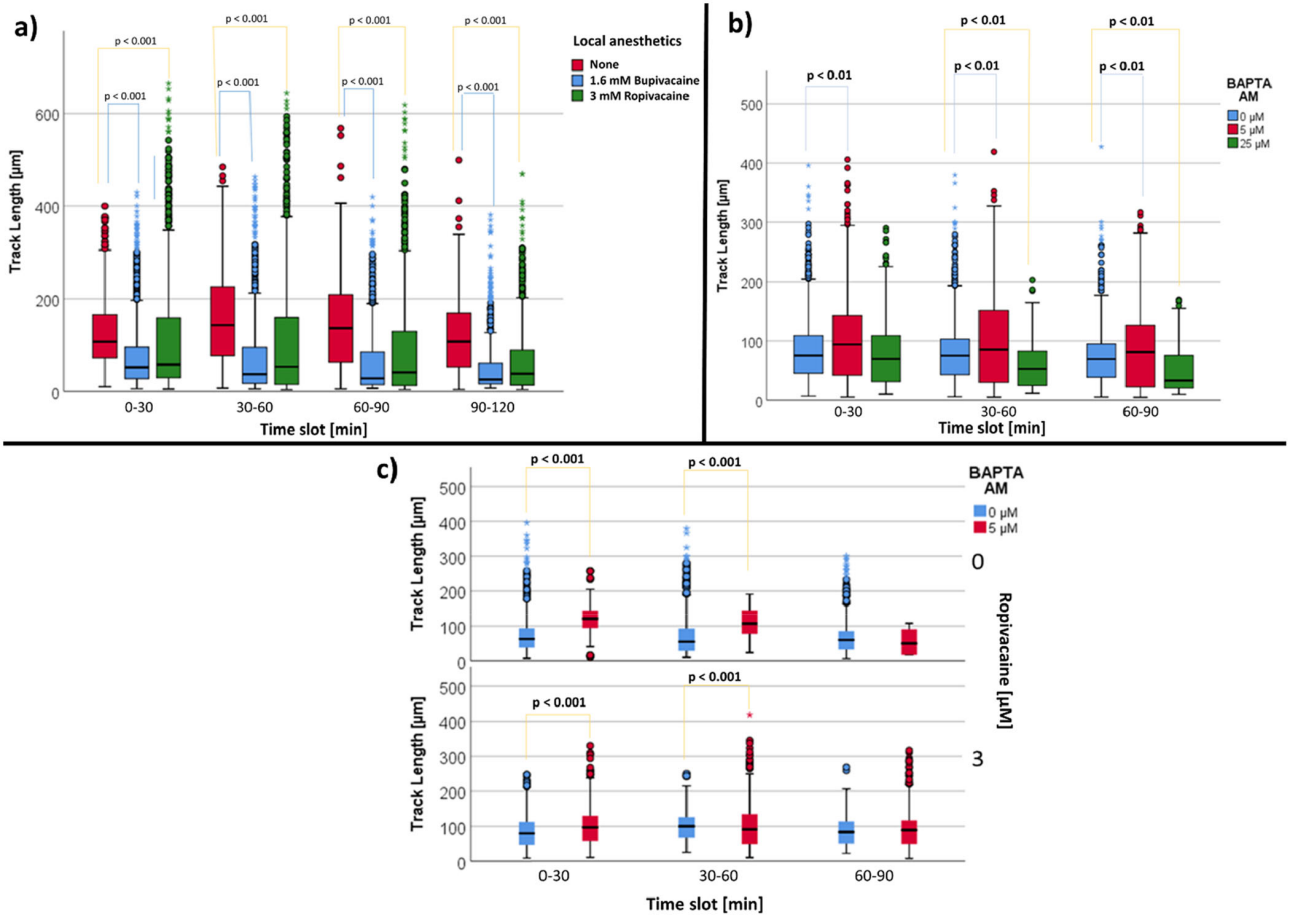
(a) Influence of bupivacaine and ropivacaine on PMN track length. Data is shown subdivided by the distinct local anesthetic. (b) Influence of BAPTA AM on PMN track length. (c) Influence of BAPTA AM and ropivacaine on PMN track length. Data is shown as median with interquartile range.

In the time slots from the experimental beginning up to 90 min oberservation time, both local anesthetics had an inhibitory effect on the PMN migration length (Figure 15a and Table S10). In the time slots up to 90 min observation time, the track length was higher with 5 µM BAPTA AM and lower with 25 µM BAPTA AM than without calcium chelator (Figure 15b and Table S11). In the first 60 min PMN track length was significantly higher in the presence of BAPTA AM (5 µM) than without BAPTA AM independent of ropivacaine presence (Figure 15c and Table S12).

#### 3.4.2 Effect of GPCR Inhibitors and Ropivacaine

**Figure 16:**
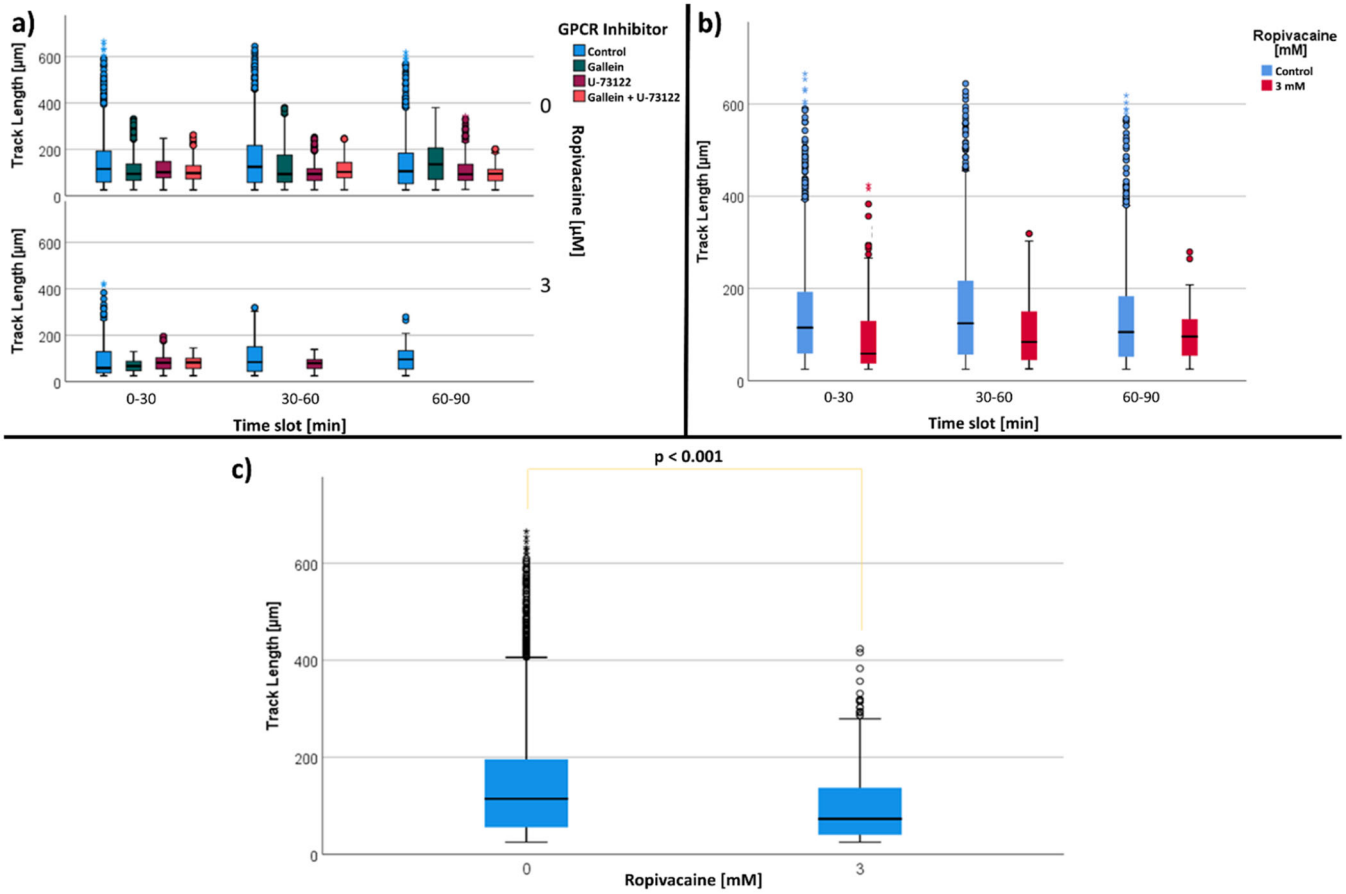
(a) Influence of GPCR inhibitors and ropivacaine on track length [µm]. Data is shown as median with interquartilrange and subdivided into GPCR inhibitors. (b) Effect of summarized Ropivacaine (3 mM) on PMN track length [µm] (c) Comparison of the influence of ropivacaine on PMN track length [µM].

The combined application of GPCR inhibitors with 3 mM ropivacaine showed an earlier cessation of cellular locomotion over time (no track lengths > 25 µm). This earlier cessation was observed (lower diagram of Figure 16a and Table S13) for experiments with Gallein and ropivacaine from time slot 30–60 min. In the time slot 60–90 min, only a small number of cells (*n* = 124 tracks) could be observed in the control group. In comparison, a higher number of cells (*n* = 366 tracks) is present at the time slot 30–60 min in presence of 3 mM ropivacaine (Figure 16a and Table S14).

The migration results of the control experiments with and without 3 mM ropivacaine were also analyzed. Since there was no clear change in the track length with increasing microscopy time in the control and with 3 mM ropivacaine (Figure 16b), the experiments were summarized and the results of the control were then compared with those using 3 mM ropivacaine, irrespective of the microscopy time. A significant difference was observed between the experiments with and without 3 mM ropivacaine (Figure 16c and Table S15).

## 4 Discussion

### 4.1 Theoretical Fundamentals for the Experimental Approach

Local anesthetics (such as bupi‐ and ropivacaine) have an impact on important PMN functions [1] and act at various sites (via GPCR and by influencing the PLC) by increasing the intracellular calcium concentration [3, 38]. The objective of our study was to investigate the effect of local anesthetics at distinct points in the signaling cascade. By performing a GPCR blockade (by Gallein) the direct effect of local anesthetics on the neutrophil GPCR could be investigated [47]. Since the local anesthetic effect on PMNs is primarily calcium‐mediated, another approach was to prevent intracellular calcium release by blocking the subsequent PLC signal cascade (by PLC inhibitor U‐73122), which is responsible for the intracellular calcium increase [45]. In the next step, released Ca^2+^ was rendered “ineffective” by the chelating agent BAPTA AM. BAPTA AM diffuses as a lipophilic substance through the PMN cell membrane and is converted by cytosolic esterases to BAPTA, which remains intracellular and binds free Ca^2+^ [50]. By using these different substances, we accomplished to investigate the impact of local anesthetics on PMN function at different sites of the signal cascade.

### 4.2 Effects of Bupivacaine and Ropivacaine on PMN Function

Various local anesthetics are able to inhibit phagocytosis, the formation of superoxides and hydrogen peroxide as well as the NETosis of PMNs in a concentration‐dependent way. These local anesthetics include lidocaine, mepivacaine, procaine and tetracaine, but also bupivacaine [51, 52]. ROS formation was described by Hann et al. as a trigger of NET formation [17]. The earlier onset of NETosis by the use of bupivacaine in clinically typical concentrations (1.58 mM to 3.16 mM) has already been demonstrated by Kolle et al. [1].

We were able to confirm these results in our study: NETosis could be triggered earlier with bupivacaine (0.1 mM to 1.6 mM) in clinically applied concentrations. In contrast, however, Mikawa et al. explained that only a 100‐fold higher concentration of lidocaine and mepivacaine than used clinically is able to reduce neutrophil ROS production and that bupivacaine showed no effect on the synthesis of ROS [53].

Ropivacaine in high doses (10 mM and 15 mM) had no significant effect on NETosis in our experiments. There was only a significant difference between the concentrations of 3 mM, 5 mM and 9 mM compared to a ropivacaine concentration of 10 mM. This is possibly due to the low number of cases with the concentrations of 10 mM and 15 mM. Thus, the effect of ropivacaine on NETosis was not bupivacaine like, but also not like that one of lidocaine in the measurements by other study groups [1]:

Blumenthal et al. were able to observe a strongly anti‐inflammatory effect on PMNs when ropivacaine was applied. This was shown by a reduction in ICAM‐1 expression and a reduction in PMN adherence. However, these observations were limited to PMNs stimulated by lipopolysaccharide [54]. Also Piegler et al. were able to demonstrate an inhibitory effect of ropivacaine on TNF‐α‐triggered inflammations, and reduce the adhesion and endothelial hyperpermeability for PMNs [55]. In contrast, Yamada et al. showed an inhibition of the formation of free oxygen radicals at low and high ropivacaine concentrations (20 µM). With increasing lipophilicity of the local anesthetic, the production of oxygen radicals was inhibited more strongly [56].

The inhibitory effect of ropivacaine on the formation of free oxygen radicals and the equally observed change in the calcium response of PMNs could also be confirmed by Mikawa et al. However, phagocytosis and migration were unaffected in these observations [57].

### 4.3 Stereoisomerism of Local Anesthetics

In everyday clinical practice, 25% of the drugs used are in racemic form. These have differences with regard to their pharmacokinetics, pharmacodynamics, and toxicity [58]. In practice, for levobupivacaine and ropivacaine S‐enantiomers are used since the R‐enantiomer has a higher adverse effect profile [59, 60]. The R‐enantiomer of bupivacaine has been described as having high toxicity [61]. It was also described that the enantiomers of the local anesthetic bupivacaine have different effects on the surface receptor expressions of PMNs, their phagocytosis and ROS production [52, 62]. Thus, it is possible in the present study to evaluate the measured effects of the local anesthetics bupivacaine and ropivacaine with regard to NETosis as a result of the different stereoisomerism.

### 4.4 Intracellular Calcium as a Trigger of NETosis

Numerous studies have described the intracellular calcium of PMNs as a prerequisite for the onset of NETosis [17, 44, 63, 64].

Vorobjeva et al., for example, triggered NETosis by fMLP. The subsequent signal transduction leads to the release of calcium stored in the ER [7]. In another study using the calcium ionophore A23187, they described that NETosis is mediated by a bacterially triggered increase in intracellular calcium. They discussed that a special form of ROS determines the induction of NETosis. This corresponds to mtROS, i.e. a A23187‐triggered ROS production that takes place in the mitochondria. Thus, an alternative way is shown here, which leads to NETosis through calcium stimulation [65]. The mechanism triggering increased mtROS production corresponds to an opening of the mitochondrial permeability transition pores (mPTP).

This NADPH oxidase‐independent NETosis was also described by Douda et al. They were able to recognize that intracellular calcium plays a key role in opening the potassium channel of the mitochondrion (SK_3_) and thus the mPTP, the activation of which is sufficient for this NOX‐independent NETosis. They also observed that, in comparison, the signaling pathways of the Akt and ERK kinases were activated in NOX‐dependent NETosis. This is not the case when using A23187 with a low activation degree of the kinases [4].

In our study, fMLP was used. This stimulates these kinases via the GPCR receptor, and activates the NADPH oxidase‐dependent signaling pathway (Figure 1) [66, 67].

### 4.5 Chelation of Intracellular Calcium

Chelation of intracellular calcium is possible with BAPTA. This could be confirmed by Parker et al. [44] and Sato et al. [68] with BAPTA AM under fMLP stimulation. Our study also aimed at chelating intracellular calcium with BAPTA AM in order to observe its effect on NETosis. The selection of the BAPTA AM concentrations was comparable to those of the study by Parker et al. [44].

In addition to the concentrations of 10 µM and 25 µM, concentrations of 5 µM, 20 µM and 30 µM were also used initially. Due to the increasing instability of the gel of the 3D µ‐slide chemotaxis chambers, the experiments with the highest concentration of 30 µM could not be evaluated. Thus, only the concentrations of 5 µM to 25 µM were used. However, our results were only partly in agreement with former studies. Compared to the controls, we observed no significant difference between the time of NETosis when BAPTA AM was used. While Gupta et al. reported that intracellular chelation of Ca^2+^ with BAPTA had no effect, Parker et al. described chelation of intracellular calcium with BAPTA‐AM to lead to a significant reduction in IL‐8 mediated NET generation, and to a lower extent in cells treated with PMA or ionomycin [44, 63].

In contrast, Kenny et al. observed calcium chelation when using BAPTA AM [69]. However, they described that the intracellularly achievable concentrations of BAPTA can no longer completely chelate calcium concentrations that are too high, as had already been observed by Gennaro et al. [70] In our study, a significant difference in IT_50_ Fluo‐4 and thus in the intracellular calcium amount could be detected compared to the control group both with simultaneous use of BAPTA AM and ropivacaine (3 mM) and with sole use of ropivacaine (3 µM).

In the experiments of our study, BAPTA AM had no effect on NETosis, which is also confirmed by the observations of Mikawa et al. They described that a suppression of the calcium concentrations could be measured with high concentrations of ropivacaine and that the PMN functions could be influenced as a result [57].

### 4.6 Surface Structure of PMNs

The activation of the GPCRS of PMNs leads to the activation of migration, ROS production and exocytosis of intracellular granules [18]. The fMLP used in our study interacts with a GPCR and thus initiates the chemotaxis of PMNs [20]. Lehmann et al. and Neptun et al. explained that GPCR signal transduction in PMNs does not occur through Gα subunits but through Gβγ subunits [22, 71].

In studies by Karuppergoundar et al. and Sanz et al., gallein was used to inhibit GPCR signal transduction, which inhibits PI3K and thus the migration of PMNs and partially their superoxide synthesis [46, 47]. Andreeva et al., however, used U‐73122 to inhibit the GPCR‐triggered PLC signaling pathway in order to inhibit superoxide synthesis [45].

In our study, the use of the GPCR inhibitors gallein and U‐73122 resulted in a significantly earlier onset of NETosis and the temporal activity of Fluo‐4 and thus of the maximum intracellular calcium activity. These effects were not observed when gallein and U‐73122 were used in parallel.

Hilger et al. described that GPCR signaling is modulated by various agonists. Thus, different effects are achieved through the binding of ligands. They also concluded that a conformational change in the GPCR would result in a better binding affinity for ligands. This would have an effect on different efficiency and kinetics in the G protein couplings [72]. The complexity of the GPCRs was also explained by Masuho et al. A GPCR consists of different compositions of Gα and Gβγ subunits. There are 16 different Gα subunits alone. As for the Gβγ complex, there are 5 different Gβ and 12 different Gγ subunits. This results in numerous combinations of the occurrence of GPCRs and influences their effectiveness [73].

The different composition of the GPCRs and their different conformations to different ligands could explain that the effect of the combined GPCR application was not measured as significant in our study.

### 4.7 GPCR Inhibitors and Local Anesthetics

A possible target of local anesthetics are ligand‐activated ion channels. Rao et al. explained that GPCRs as membrane proteins in the lipid bilayer play a role in the action of local anesthetics [74]. The efficiency of GPCR signal transduction can be modulated by their own dynamics, and their interaction can be regulated in the membrane [75, 76]. The lateral diffusion of membrane proteins and thus of GPCRs plays an important role in signal transduction [75]. An important factor that limits the lateral diffusion in the biological membrane is the actin cytoskeleton beneath the plasma membrane [77]. Furthermore, Rao et al. concluded from their study that the receptor dynamics of GPCRs and their interaction with the actin cytoskeleton can be relevant factors for the action mechanism of local anesthetics [74]. Despite all that, the use of the local anesthetic ropivacaine in our study showed no additional significant effect on the earlier onset of NETosis and on intracellular calcium.

### 4.8 Effects of Local Anesthetics on Migration

The fact that local anesthetics can have an inhibitory effect on migration could already be demonstrated in previous studies [1, 52, 78, 79]. Also our study observed an earlier termination of migration when bupivacaine (1.6 mM) and ropivacaine (3 mM) were used.

### 4.9 Effects of BAPTA AM on Migration

Parker et al. [44] and Gupta et al. [63] confirm that the intracellular calcium of PMNs is a crucial factor for triggering migration. With our tests, we were able to confirm that chelation of intracellular calcium with BAPTA AM leads to an earlier onset of migration compared to the control group. This indicates that intracellular calcium is an important component of the signaling pathways for migration. A significant effect of ropivacaine in combination with BAPTA AM could not be measured.

### 4.10 Inhibition of Migration by GPCR Inhibitors

Surve et al. have already demonstrated that the Gβγ subunit of the GPCR is crucial for the chemotaxis of PMNs and their migration [23]. Likewise, Dixit et al. were able to show that the GPCR signaling pathway plays a significant role in enhancing the adhesion of PMNs and triggering their migration [80]. When using the GPCR inhibitors gallein or PLC inhibitor U‐73122, our study observed an earlier suspension of migration and maximum intracellular calcium level. However, the result could not be significantly evaluated because different cell numbers were present at the different time points detected. However, these results are in agreement with the observations by Surve et al. and Dixit et al.

### 4.11 Pharmacokinetic Considerations

The pharmacokinetics of ropivacaine are contingent on several factors, including the administered dose, the route of administration, the time required to reach the desired blood concentration, and the patient′s comorbidities [81, 82]. The absorption of bupivacaine is also dose‐dependent, whereby the pharmacokinetic properties of levobupivacaine are similar to that of bupivacaine [82].

There is a dose–response relationship between the plasma concentration of a local anesthetic and its systemic toxicity. However, this relationship is neither simple nor monotonic, and it depends on a number of factors, including the rate of change of plasma concentration.

For example, the absorption of bupivacaine is biphasic after epidural application, with a small amount of the drug being rapidly absorbed, followed by a slower absorption of the remainder [82]. Depending on the application method, haemodynamics and the vascularization at the application site, ropi‐ and bupivacaine bind to plasma proteins. It is the non‐protein bound fraction of the local anesthetics that is active and factors that influence the degree of protein binding will alter its toxicity [82].

The concentration of the local anesthetic present in the aqueous portion of plasma is directly related to tissue absorption, and hence toxicity. Maximum plasma concentration is therefore related to the apparent toxicity of local anesthetics, with highly aerobic tissues, such as the myocardium, CNS and lung, being most vulnerable to toxic effects [82].

In the context of neuro‐ and (heart)muscle toxicity, pathophysiological studies have identified increased intracellular Ca²⁺ levels as a critical factor in neuronal and (cardio)myocyte injury. To a different extent, bupi‐ and ropivacaine in clinical concentrations induce Ca^2+^ release from the sarcoplasmic reticulum and simultaneously inhibit Ca^2+^ reuptake into the sarcoplasmic reticulum, resulting in cytotoxic intracellular Ca^2+^ levels [83]. The effect of the local anesthetic on the PMNs is also primarily calcium‐mediated [50]. It is this juncture, where BAPTA AM can intervene. It showed a cell‐ and neuroprotective effect even at low concentration (3 µM), though accompanied by disturbed calcium homeostasis [84].

BAPTA AM it is generally presumed to be converted to BAPTA through enzymatic cleavage of the acetoxymethyl ester linkages by carboxylesterase based on the indirect evidence such as the change of pH value. Due to the rapid hydrolysis of BAPTA‐AM by plasma carboxylesterase, BAPTA‐AM plasma concentration–time profile could not support a thorough pharmacokinetic evaluation. On the contrary, BAPTA was stable and the concentration was relatively high in rat plasma. However, the fecal excretion of BAPTA was the major elimination pathway of BAPTA‐AM [85]. The elimination pathway is similar to bupi‐ and ropivacaine, which initially involves the metabolism of inactive metabolites by cytochromes, primarily CYP1A2 and CYP3A, and then excreted in urine and feces [81, 82].

In patients with heart failure, treatment with Gallein alleviates cardiac dysfunction, fibrosis and the infiltration of inflammatory (macrophages) and mast cells [46]. Thereby, binding and dissociation rates for Gallein to GPCR were relatively low (half‐time value 3000 s = 50 min). This is possibly associated with a slow onset of action, but also a prolonged duration of action with an additional protective mechanism [22]. Contrary, addition of U73122 resulted in a rapid decline of intracellular Ca^2+^ levels with marked attenuation of the prolonged plateau phase, suggesting that persistent PLC activity is primarily responsible for exaggerated intracellular Ca^2+^ release and in our study for the influenced PMN migration [86].

## 5 Conclusion

The local anesthetics bupivacaine and ropivacaine exert a significant effect on neutrophil migration and NETosis. Intracellular calcium does not seem to be crucial for NETosis, but very important for neutrophil migration. The inhibition of the Gβγ subunit with Gallein and U‐73122 resulted in an earlier onset of NETosis and maximum intracellular calcium concentration in PMNs. However, an additional effect of the local anesthetic ropivacaine on the GPCR signaling pathway could not be found. Future research will be necessary for a more detailed understanding of the signal transduction pathways in PMNs. This is essential for being able to achieve a targeted influence on neutrophil functions.

## Author Contributions

**Richard Felix Kraus:** writing – original draft, visualization, validation, investigation, methodology, software, data curation, formal analysis, resources. **Thies Galla:** investigation, methodology, validation, visualization, formal analysis. **Michael Gruber:** writing – review and editing, conceptualization, funding acquisition, investigation, project administration, data curation, resources. **Sigrid Wittmann:** writing – review and editing, conceptualization, methodology, supervision, data curation, project administration.

## Conflicts of Interest

The authors declare no conflicts of interest.

## Transparency Statement

The lead author Richard Kraus affirms that this manuscript is an honest, accurate, and transparent account of the study being reported; that no important aspects of the study have been omitted; and that any discrepancies from the study as planned (and, if relevant, registered) have been explained.

## Supporting information

Declaration dual publication.

supmat.

## Data Availability

The data that support the findings of this study are available on request from the corresponding author. The data are not publicly available due to privacy or ethical restrictions.
