# Supplementary material for: Effect of Ropivacain and Bupivacain on Calcium‐Related and G‐Protein Coupled Processes in PMNs: A Human In‐Vitro Study
Source: Health Sci Rep. 2025 Dec 9;8(12):e71636. doi: 10.1002/hsr2.71636 (PMC12689273; doi:10.1002/hsr2.71636)
Supplement: Supplementary file 1 — Declaration dual publication. [file HSR2-8-e71636-s001.docx]

Some results on which Figure 9b is based have already been published by other members of our research group (Sixt et al., 2023). Nevertheless, different parameters and contexts were considered: The data presented in both studies are analyzed and represented using different parameters. In Sixt et al., the focus is on EC50 values plotted against local anesthetic concentrations, while in our work, the emphasis is on ET50 values related to the time of the half-maximal effect. This difference in the parameters used indicates that the studies are addressing different aspects of the data.

References

Sixt, S., M. Gruber, G. Kolle, T. Galla, and D. Bitzinger. 2023. The Effect of Local Anesthetics on Neutrophils in the Context of Different Isolation Techniques. *Biomedicines* 11. doi:10.3390/biomedicines11082170.
