# Supplementary material for: Effect of Ropivacain and Bupivacain on Calcium‐Related and G‐Protein Coupled Processes in PMNs: A Human In‐Vitro Study
Source: Health Sci Rep. 2025 Dec 9;8(12):e71636. doi: 10.1002/hsr2.71636 (PMC12689273; doi:10.1002/hsr2.71636)
Supplement: Supplementary file 2 — supmat. [file HSR2-8-e71636-s002.docx]

Supplement

## Influence of Bupivacaine on ET_50_NETosis

Table S 1: Mean and standarddeviation of parameter ET_50_NETosis in presence of bupivacaine (data illustrated in Figure 13a)

| Concentration [mM] | Mean [min] | Standard Deviation [min] | Number of experiments (n) |
| --- | --- | --- | --- |
| 0.0 - 0.1 | 331.0 | 135.5 | 9 |
| 0.2 - 0.5 | 432.3 | 124.3 | 5 |
| 1.6 | 115.7 | 14.6 | 3 |

## Influence of Ropivacaine on ET_50_NETosis

Table S 2: Mean and standarddeviation of parameter ET_50_NETosis in presence of ropivacaine (data illustrated in Figure 13b)

| Concentration [mM] | Mean [min] | Standard Deviation [min] | Number of experiments (n) |
| --- | --- | --- | --- |
| 0.0 | 311.8 | 162.3 | 29 |
| 0.1 | 526.3 | 413.9 | 7 |
| 0.5 | 508.8 | 9.1 | 8 |
| 1.0 | 505.9 | 194.9 | 7 |
| 3.0 | 270.5 | 143.2 | 33 |
| 5.0 | 275.1 | 244.4 | 5 |
| 9.0 | 210.4 | 22.9 | 4 |
| 10.0 | 455.0 | 101.0 | 5 |
| 15.0 | 359.7 | 63.1 | 2 |

## Influence of Ropivacaine on ET_50_NETosis

Table S 3: Median and interquartile ranges of parameter ET_50_NETosis in presence of ropivacain with combined concentrations (data illustrated in Figure 13c)

| Concentration [mM] | Median [min] | Interqartile Range [min] | Number of experiments (n) |
| --- | --- | --- | --- |
| 0 | 234.8 | 233.6 | 29 |
| 0.1-1.0 | 372.3 | 269.0 | 22 |
| 3.0 – 15.0 | 267.8 | 166.1 | 49 |

## Influence of BAPTA-AM, GPCR inhibitors and Ropivacaine neutrophil NETosis

Table S 4: Median and interquartile ranges of parameter ET_50_NETosis after addition of BAPTA-AM combined with presence of ropivacaine (data illustrated in Figure 14a)

| GPCR inhibitor | Median [min] | Interqartile Range [min] | Number of experiments (n) |
| --- | --- | --- | --- |
| 0▒µM BAPTA AM/  0▒µM ropivacaine | 414.4 | 246.2 | 16 |
| 0▒µM BAPTA AM/  3▒mM ropivacaine | 300.4 | 271.2 | 11 |
| <25▒µM BAPTA AM/  0▒µM ropivacaine | 267.2 | 118.4 | 8 |
| <25▒µM BAPTA AM/  3▒mM ropivacaine | 268.9 | 106.5 | 13 |

Table S 5: Median and interquartile ranges of parameter IT50Fluo after addition of BAPTA-AM combined with presence of ropivacaine (data illustrated in Figure 14b)

| GPCR inhibitor | Median [min] | Interqartile Range [min] | Number of experiments (n) |
| --- | --- | --- | --- |
| 0▒µM BAPTA AM/  0▒µM ropivacaine | 410.7 | 150.0 | 17 |
| 0▒µM BAPTA AM/  3▒mM ropivacaine | 281.6 | 221.6 | 11 |
| <25▒µM BAPTA AM/  0▒µM ropivacaine | 302.1 | 120.6 | 7 |
| <25▒µM BAPTA AM/  3▒mM ropivacaine | 262.4 | 57.4 | 12 |

Table S 6: Median and interquartile ranges of parameter ET_50_NETosis after addition of GPCR inhibitors (data illustrated in Figure 14c)

| GPCR inhibitor | Median [min] | Interqartile Range [min] | Number of experiments (n) |
| --- | --- | --- | --- |
| Control | 415.3 | 189.3 | 14 |
| Gallein | 171.3 | 74.9 | 8 |
| U-73122 | 198.4 | 36.7 | 8 |
| Gallein + U 73122 | 250.1 | 133.9 | 8 |

Table S 7: Median and interquartile ranges of parameter IT50Fluo after addition of GPCR inhibitors (data illustrated in Figure 14▒d)

| GPCR inhibitor | Median [min] | Interqartile Range [min] | Number of experiments (n) |
| --- | --- | --- | --- |
| Control | 418.7 | 122.6 | 22 |
| Gallein | 267.0 | 186.7 | 5 |
| U-73122 | 252.0 | 93.7 | 5 |
| Gallein + U 73122 | 286.2 | 225.4 | 5 |

Table S 8: Median and interquartile ranges of parameter ET50NETosis after addition of GPCR inhibitors combined with presence of ropivacaine (data illustrated in Figure 14e)

| GPCR inhibitor | Median [min] | Interqartile Range [min] | Number of experiments (n) |
| --- | --- | --- | --- |
| Control + ropivacaine | 417.0 | 270.8 | 66 |
| Gallein + ropivacaine | 171.3 | 74.9 | 8 |
| U-73122 + ropivacaine | 198.4 | 36.7 | 8 |
| Gallein + U 73122 + ropivacaine | 250.1 | 133.9 | 8 |

Table S 9: Median and interquartile ranges of parameter IT_50_Fluo after addition of GPCR inhibitors combined with presence of ropivacaine (data illustrated in Figure 14▒f)

| GPCR inhibitor | Median [min] | Interqartile Range [min] | Number of experiments (n) |
| --- | --- | --- | --- |
| Control + ropivacaine | 404.0 | 185.0 | 69 |
| Gallein + ropivacaine | 228.4 | 70.3 | 10 |
| U-73122 + ropivacaine | 245.1 | 62.7 | 10 |
| Gallein + U 73122 + ropivacaine | 282.0 | 154.0 | 8 |

## Influence of Bupi- and Ropivacaine on neutrophil Migration

Table S 10: Influence of bupivacain (n▒=▒17) and ropivacain (n▒=▒41) on track length [µm]. Data is shown as median with interquartilrange and subdivided into 30▒min time slots (data is illustrated in Figure 15a)

| Time  slot | | Local anestethics | Mean  [min] | Standard Deviation [min] | Number of experiments (µSlide channels) | Cell number  (n) |
| --- | --- | --- | --- | --- | --- | --- |
| 0-30 | Control | | 122.3 | ± 73.3 | 29 | 761 |
|  | Bupivacaine | | 75.0 | ± 71.1 | 17 | 1397 |
|  | Ropivacaine | | 106.6 | ± 112.0 | 41 | 1872 |
| 30-60 | Control | | 156.0 | ± 98.9 | 29 | 537 |
|  | Bupivacaine | | 78.0 | ± 93.8 | 17 | 741 |
|  | Ropivacaine | | 101.1 | ± 112.5 | 41 | 1706 |
| 60-90 | Control | | 605 | ± 96.0 | 29 | 605 |
|  | Bupivacaine | | 68.9 | ± 82.3 | 17 | 596 |
|  | Ropivacaine | | 82.1 | ± 93.2 | 41 | 1804 |
| 90-120 | Control | | 116.1 | ± 80.5 | 29 | 617 |
|  | Bupivacaine | | 52.4 | ± 63.0 | 17 | 632 |
|  | Ropivacaine | | 62.1 | ± 64.8 | 41 | 1745 |

## Influence of BAPTA AM and Ropivacaine on neutrophil Migration

Table S 11: Influence of different concentrations of BAPTA AM on track length subdivided in different 30▒min time slots [µm]. Data is shown as median with interquartilrange (data is illustrated in Figure 15b)

| Time slot | c (BAPTA AM) [mM] | Mean  [min] | | Standard Deviation [min] | Number of experiments (µSlide channels) | Cell number  (n) |
| --- | --- | --- | --- | --- | --- | --- |
| 0-30 | 0 | 83.5 | | ± 50.8 | 33 | 2580 |
|  | 5 | 103.1 | | ± 71.8 | 5 | 1133 |
|  | 25 | 83.1 | | ± 63.4 | 4 | 308 |
| 30-60 | 0 | 79.9 | | ± 49.4 | 33 | 2142 |
|  | 5 | 102.2 | ± 79.2 | | 5 | 899 |
|  | 25 | 58.6 | ± 38.9 | | 4 | 280 |
| 60-90 | 0 | 73.2 | ± 47.4 | | 33 | 1385 |
|  | 5 | 86.1 | ± 64.3 | | 5 | 1046 |
|  | 25 | 49.0 | ± 37.0 | | 4 | 220 |

Table S 12: Influence of BAPTA AM and ropivacaine on track length [µm]. Data is shown as median with interquartilrange and subdivided into 30▒min time slots (data is illustrated in Figure 15c)

| c (ropivacaine) [mM] | Time  slot | c (BAPTA AM) [mM] | | Mean  [min] | Standard Deviation [min] | | Number of experiments (µSlide channels) | Cell number  (n) |
| --- | --- | --- | --- | --- | --- | --- | --- | --- |
| 0 | 0-30 | | 0 | 75.4 | ± 54.5 | 9 | | 728 |
|  |  |  | 5 | 122.5 | ± 53.1 | 4 | | 144 |
|  | 30-60 | | 0 | 73.5 | ± 69.9 | 9 | | 489 |
|  |  |  | 5 | 110.0 | ± 46.7 | 4 | | 40 |
|  | 60-90 | | 0 | 69.2 | ± 52.8 | 9 | | 497 |
|  |  |  | 5 | 54.3 | ± 32.6 | 4 | | 22 |
| 3.0 | 0-30 | | 0 | 84.8 | ± 47.3 | 10 | | 738 |
|  |  |  | 5 | 102.9 | ± 58.3 | 6 | | 492 |
|  | 30-60 | | 0 | 101.0 | ± 43.9 | 10 | | 304 |
|  |  |  | 5 | 101.8 | ± 70.3 | 6 | | 371 |
|  | 60-90 | | 0 | 88.3 | ± 47.4 | 10 | | 131 |
|  |  |  | 5 | 93.8 | ± 62.8 | 6 | | 434 |

## Influence of GPCR inhibitors and Ropivacaine on neutrophil Migration

Table S 13: Influence of GPCR inhibitors and ropivacaine on track length [µm]. Data is shown as median with interquartilrange and subdivided into GPCR inhibitors (data is illustrated in Figure 16a)

| c (ropivacaine) [mM] | Time  slot | | GPCR  Inhibitor | Mean  [min] | Standard Deviation [min] | | Number of experiments (µSlide channels) | Cell number  (n) |
| --- | --- | --- | --- | --- | --- | --- | --- | --- |
| 0 | 0-30 | Control | | 140.7 | | ± 103.8 | 23 | 2345 |
|  |  | U-73122 | | 111.2 | | ± 47.2 | 4 | 696 |
|  |  | Gallein | | 110.6 | | ± 65.9 | 4 | 645 |
|  |  | Gallein +  U-73122 | | 103.6 | | ± 45.4 | 7 | 1455 |
|  | 30-60 | Control | | 150.3 | | ± 109.8 | 23 | 2007 |
|  |  | U-73122 | | 98.9 | | ± 47.8 | 4 | 506 |
|  |  | Gallein | | 126.3 | | ± 90.1 | 4 | 572 |
|  |  | Gallein +  U-73122 | | 112.5 | | ± 52.4 | 7 | 500 |
|  | 60-90 | Control | | 129.0 | | ± 93.7 | 23 | 2208 |
|  |  | U-73122 | | 111.6 | | ± 69.3 | 4 | 286 |
|  |  | Gallein | | 150.0 | | ± 90.0 | 4 | 354 |
|  |  | Gallein +  U-73122 | | 92.7 | | ± 37.1 | 7 | 451 |
| 3.0 | 0-30 | Control | | 91.2 | | ± 73.6 | 12 | 366 |
|  |  | U-73122 | | 82.0 | | ± 33.6 | 6 | 944 |
|  |  | Gallein | | 68.3 | | ± 25.1 | 6 | 542 |
|  |  | Gallein +  U-73122 | | 79.4 | | ± 27.5 | 6 | 720 |
|  | 30-60 | Control | | 104.8 | | ± 72.1 | 12 | 194 |
|  |  | U-73122 | | 75.8 | ± 25.8 | | 6 | 363 |
|  | 60-90 | Control | | 97.6 | ± 53.5 | | 12 | 124 |

## Influence of Ropivacaine on neutrophil Migration

Table S 14: Influence of ropivacaine on track length [µm] subdivided in 30▒min time slots. Data is shown as median with interquartilrange (data is illustrated in Figure 16b)

| c (ropivacaine) [mM] | Time  slot | Mean  [min] | | Standard Deviation [min] | Number of experiments (µSlide channels) | | | Cell number  (n) |
| --- | --- | --- | --- | --- | --- | --- | --- | --- |
| 0 | 0-30 | 140.7 | | ± 103.8 | 39 | | | 2345 |
|  | 30-60 | 150.3 | | ± 109.8 | 39 | | | 2007 |
|  | 60-90 | 129.0 | | ± 93.7 | 39 | | | 2208 |
| 3.0 | 0-30 | 91.2 | | ± 73.6 | 33 | | | 366 |
|  | 30-60 | 104.8 | ± 72.1 | | | 33 | 194 | |
|  | 60-90 | 97.6 | ± 53.5 | | | 33 | 124 | |

Table S 15: Influence of ropivacaine on track length [µm]. Data is shown as median with interquartilrange (data is illustrated in Figure 16c)

| c (ropivacaine) [mM] | Mean  [min] | Standard Deviation [min] | Number of experiments (µSlide channels) | Cell number  (n) |
| --- | --- | --- | --- | --- |
| 0 | 139.7 | ± 102.8 | 39 | 6560 |
| 3.0 | 96.2 | ± 70.1 | 33 | 684 |
